# Neuropilin-1 Mediates SARS-CoV-2 Infection of Astrocytes in Brain Organoids, Inducing Inflammation Leading to Dysfunction and Death of Neurons

**DOI:** 10.1128/mbio.02308-22

**Published:** 2022-10-31

**Authors:** Weili Kong, Mauricio Montano, Michael J. Corley, Ekram Helmy, Hirofumi Kobayashi, Martin Kinisu, Rahul Suryawanshi, Xiaoyu Luo, Loic A. Royer, Nadia R. Roan, Melanie Ott, Lishomwa C. Ndhlovu, Warner C. Greene

**Affiliations:** a Michael Hulton Center for HIV Cure Research at Gladstone, San Francisco, California, USA; b Gladstone Institute of Virology, San Francisco, California, USA; c Department of Medicine, University of California, San Francisco, San Francisco, California, USA; d Department of Microbiology and Immunology, University of California, San Francisco, San Francisco, California, USA; e Department of Urology, University of California, San Francisco, San Francisco, California, USA; f Division of Infectious Diseases, Department of Medicine, Weill Cornell Medicine, New York, New York, USA; g CZ Biohub, San Francisco, California, USA; McMaster University

**Keywords:** COVID-19, astrocytes, innate immunity, inflammation, neuronal dysfunction, human iPSC-derived brain organoids

## Abstract

Coronavirus disease 2019 (COVID-19) is frequently associated with neurological deficits, but how severe acute respiratory syndrome coronavirus 2 (SARS-CoV-2) induces these effects remains unclear. Here, we show that astrocytes are readily infected by SARS-CoV-2, but surprisingly, neuropilin-1, not angiotensin-converting enzyme 2 (ACE2), serves as the principal receptor mediating cell entry. Infection is further positively modulated by the two-pore segment channel 2 (TPC2) protein that regulates membrane trafficking and endocytosis. Astrocyte infection produces a pathological response closely resembling reactive astrogliosis characterized by elevated type I interferon (IFN) production, increased inflammation, and the decreased expression of transporters of water, ions, choline, and neurotransmitters. These combined events initiated within astrocytes produce a hostile microenvironment that promotes the dysfunction and death of uninfected bystander neurons.

## INTRODUCTION

While primary infection by severe acute respiratory syndrome coronavirus 2 (SARS-CoV-2) primarily involves the respiratory tract and can lead to coronavirus disease 2019 (COVID-19), infection and damage may extend to other organs, including the brain, heart, kidney, liver, and gut (1–3). End-stage COVID-19 often presents with multiorgan failure (1, 4). In terms of brain-associated symptoms, loss of smell and taste, headache, seizures, confusion, and/or acute psychosis can occur (5–7). Furthermore, as many as 1 in 2 COVID-19 patients exhibit persistent symptoms 3 to 6 months after the acute infection, a condition termed postacute sequelae of COVID-19 (PASC) (8). Neurological manifestations of PASC include continued loss of taste and smell, “brain fog,” fatigue, and headaches (9, 10). PASC severity ranges from mild to completely debilitating and can involve multiple organ systems (11).

Low levels of SARS-CoV-2 RNA and antispike antibodies (Abs) have been reported in both the cerebrospinal fluid (CSF) and brain tissue of COVID-19 patients (12–16), suggesting that pathological changes may be caused by direct infection of key cells in the brain. Of note, it has been difficult to identify live virus in these brain tissues. Conversely, other studies have found evidence of inflammation and fibrin deposition in the apparent absence of direct virus infection in the brain (17, 18). Together, these findings suggest that the effects of SARS-CoV-2 on the brain may involve both direct and indirect mechanisms. However, whether direct or indirect, significant loss of brain gray matter occurs in some severe cases (19), emphasizing the serious and likely irreversible neuropathic potential of SARS-CoV-2.

Astrocytes control several steps of brain development and homeostasis. These cells support the formation, maturation, and maintenance of synapses via pruning and remodeling activities. These functions decline with increasing age and are undermined in various neurological diseases (20, 21). Astrocytes control the release and reuptake of neurotransmitters and the production of trophic factors pivotal for neuronal differentiation and survival. Abnormal astrocyte function impairs neuronal survival and promotes neurodegeneration (22, 23). Astrocytes from both hamsters and brain organoids have been shown to be susceptible to SARS-CoV-2 infection (15, 24, 25). Given the essential role of astrocytes in neuronal health and synapse function, it is possible that the neurological symptoms of COVID-19 result in part from the direct infection of astrocytes by SARS-CoV-2.

Direct infection requires the ability of the virus to gain entry into the central nervous system (CNS) and then bind and fuse to one or more brain cell types. Recent studies have described SARS-CoV-2 infection of neural progenitor cells (NPCs) and neurons leading to an abnormal distribution of the tau protein within neuronal cells (16, 26, 27). Four studies report marked infection of astrocytes in the brain, but these cells express extremely low levels of angiotensin-converting enzyme 2 (ACE2) (15, 25, 28, 29). The low expression level of ACE2 in CNS cells led us to hypothesize that an alternative receptor is responsible for SARS-CoV-2 infection in the CNS.

Our studies focus on four major questions: (i) what receptor(s) mediates the entry of SARS-CoV-2 into astrocytes that poorly express ACE2; (ii) do different variants of SARS-CoV-2 display different abilities to infect astrocytes; (iii) how does SARS-CoV-2 infection alter astrocyte gene expression measured by transcriptome sequencing (RNA-seq), DNA methylation profiling, and NanoString spatial profiling; and (iv) how do these transcriptomic changes impact neuronal function and survival? For these studies, we have employed both induced pluripotent stem cell (iPSC)-derived brain organoids and cultures of primary human astrocytes.

## RESULTS

### Astrocytes are permissive to SARS-CoV-2 infection.

**FIG 1:**
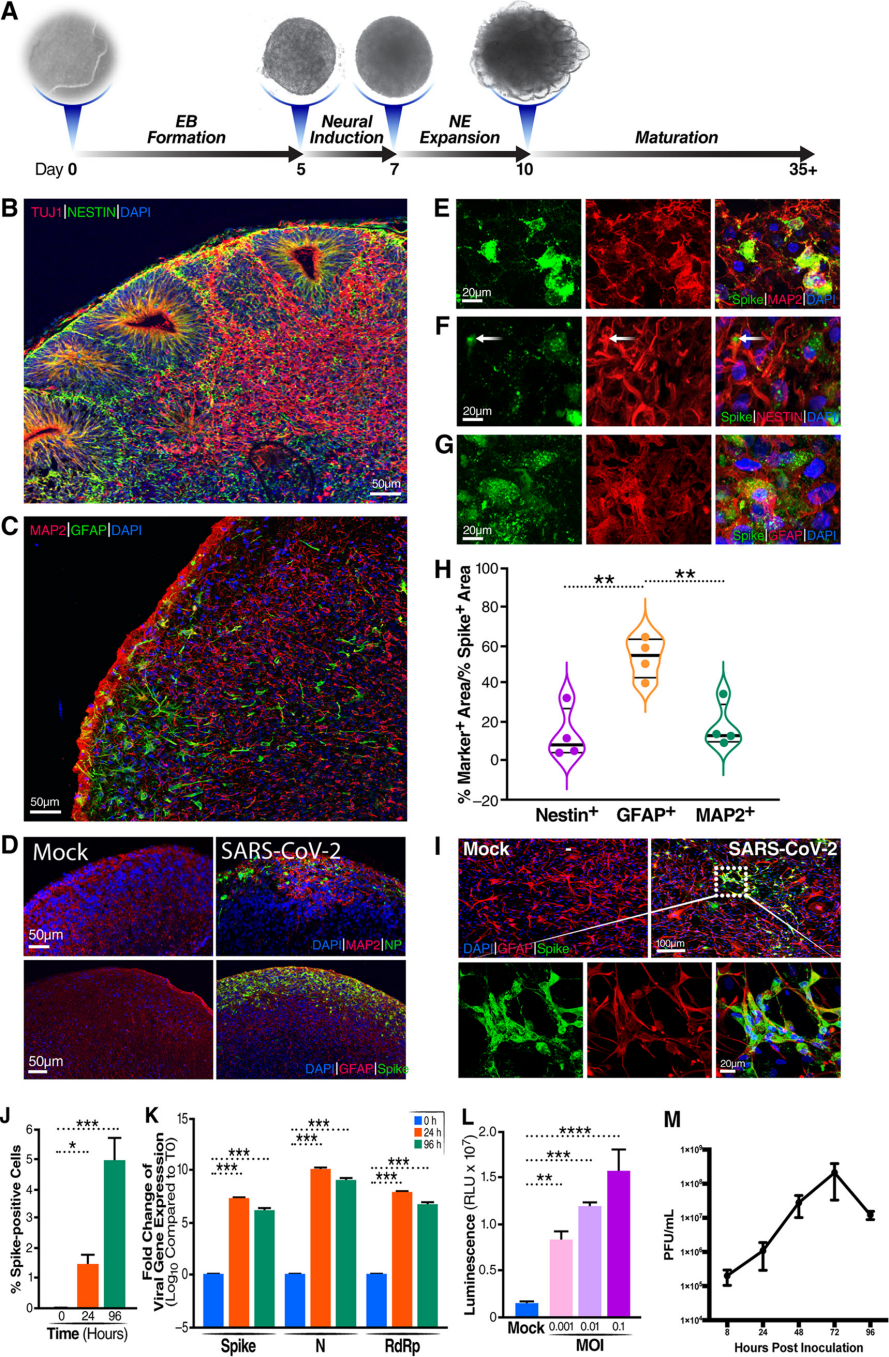
SARS-CoV-2 infects astrocytes present in brain organoids and two-dimensional cultures of primary human astrocytes. (A) Schematic overview of the procedures for generating cerebral organoids from human iPSCs using the STEMdiff cerebral organoid kit NE, Neuroepithelial. (B) Representative images of the ventricular zone-like structure formed by newborn neurons (TUJ1) (red) and neural progenitor cells (NESTIN) (green) in cerebral organoids at week 4. (C) Confocal images demonstrating the presence of astrocytes (GFAP) (green) and neurons (MAP2) (red) within the brain organoids at week 8. (D) Four-month-old cerebral organoids were either mock infected or infected with SARS-CoV-2 (WA1) at a multiplicity of infection (MOI) of 1 for 7 days. After 6 h of incubation, the medium was replaced. Shown is immunofluorescence staining for neurons (MAP2) or astrocytes (GFAP) and SARS-CoV-2 NP- or spike-positive cells (green). 4′,6-Diamidino-2-phenylindole (DAPI) was used to stain double-stranded DNA. (E) Neurons are also infected with SARS-CoV-2, as indicated by colabeling with MAP2 and viral spike in brain organoids. (F) SARS-CoV-2 infects neural progenitor cells, as indicated by the costaining of cells with NESTIN (NPC marker) and spike in brain organoids. (G) Astrocytes are infected by SARS-CoV-2, as indicated by colabeling with GFAP and viral spike in brain organoids. (H) Quantification of immunostaining data reveals significantly higher levels of SARS-CoV-2 infection of astrocytes than of neurons and neural progenitor cells (*n* = 4 sections from four organoids from two independent experiments). *, *P* < 0.05; **, *P* < 0.01 (by Student’s two-tailed *t* test). (I) Primary cultures of human astrocytes were infected with SARS-CoV-2 and immunostained 4 days later. SARS-CoV-2 spike (green), GFAP (red), and DAPI staining of cell nuclei (blue) are shown. (J) Astrocytes were infected with SARS-CoV-2 at an MOI of 0.01, and after 2 h of incubation, the medium was replaced to deplete residual virions. The percentages of SARS-CoV-2 spike-positive cells present in infected astrocyte cultures at 0, 24, and 96 hpi were assessed. Four image fields per group were quantitated for the presence of spike. *, *P* < 0.05; ***, *P* < 0.001 (using Student’s two-tailed *t* test). Error bars indicate SEM. (K) Astrocytes were infected with SARS-CoV-2 at an MOI of 0.01, and after 2 h of incubation, the medium was completely replaced. Fold increases in viral spike, N, and RdRp define RNA levels assessed by RT-qPCR in SARS-CoV-2-infected astrocytes measured at 0 hpi (T0), 24 hpi, and 96 hpi. *, *P* < 0.05; **, *P* < 0.01; ***, *P* < 0.001 (by one-way ANOVA). Results depict results from three independent experiments performed in triplicate; error bars indicate SEM. (L) Human astrocytes were infected for 48 h with SARS-CoV-2-Nluc at an MOI of 0.001, 0.01, or 0.1. Luciferase activity was measured after cell harvest. *, *P* < 0.05; **, *P* < 0.01; ***, *P* < 0.001; ****, *P* < 0.0001 (by one-way ANOVA). Data from a representative experiment from three independent experiments performed in triplicate are presented. Error bars reflect SEM. RLU, relative luciferase units. (M) Replication of SARS-CoV-2 in astrocyte cells infected with the indicated viruses at an MOI of 0.1. After 2 h of incubation, the medium was replaced. The supernatants were collected at the indicated time points and concentrated. Viral titers present in the supernatant samples were determined by plaque assays performed with Vero-E6 cells.

To better understand the neurotropism of SARS-CoV-2, we generated brain organoids using human iPSCs according to a protocol established previously by Lancaster and Knoblich (Fig. 1A to C) (30). Due to the limitations of this protocol, there are no microglial cells in this brain model. Four-month-old brain organoids were infected with SARS-CoV-2 at a multiplicity of infection (MOI) of 1 for 1 week, followed by immunostaining for the expression of viral spike and nucleocapsid protein (NP). Both SARS-CoV-2 NP and spike were detected in virally exposed but not mock-treated brain organoids (Fig. 1D). Glial fibrillary acidic protein (GFAP) staining, which marks astrocytes, sharply colocalized with spike staining, suggesting that SARS-CoV-2 targets astrocyte infection in these brain organoids (Fig. 1G and H). We also found that SARS-CoV-2 is able to infect neurons and neural progenitor cells at lower levels, as demonstrated by the colocalization of MAP2 with spike and NESTIN, respectively (Fig. 1E to H). Together, these data suggest that high levels of SARS-CoV-2 infection remain in astrocytes, although lower levels of infection in neurons and neural progenitor cells also occur.

To confirm viral infection of astrocytes, we assessed whether primary astrocytes isolated from the human cortex can be productively infected by SARS-CoV-2. Spike expression was readily detected in GFAP-positive (GFAP^+^) cells, confirming SARS-CoV-2 infection of astrocytes (Fig. 1I). By 4 days postinfection (dpi), 2% to 6% of the GFAP^+^ cells were positive for spike (Fig. 1J). Reverse transcription-quantitative PCR (RT-qPCR) experiments also revealed elevated levels of spike, viral N, and RNA-dependent RNA polymerase (RdRp) mRNAs within the SARS-CoV-2-exposed astrocytes (Fig. 1K). As expected, increasing the MOI of an infectious SARS-CoV-2 clone containing a NanoLuc reporter (SARS-CoV-2-Nluc) resulted in increased luciferase signals in astrocytes (Fig. 1L). In parallel, we evaluated the productive replication of SARS-CoV-2 in astrocytes by measuring infectious titers from the supernatants collected at 8, 24, 48, 72, and 96 h postinfection (hpi) by plaque assays. Viral titers steadily increased, peaking at 72 hpi (Fig. 1M). These results support the conclusion that astrocytes can support SARS-CoV-2 replication.

### Neuropilin-1 functions as the primary receptor for SARS-CoV-2 entry into astrocytes.

**FIG 2:**
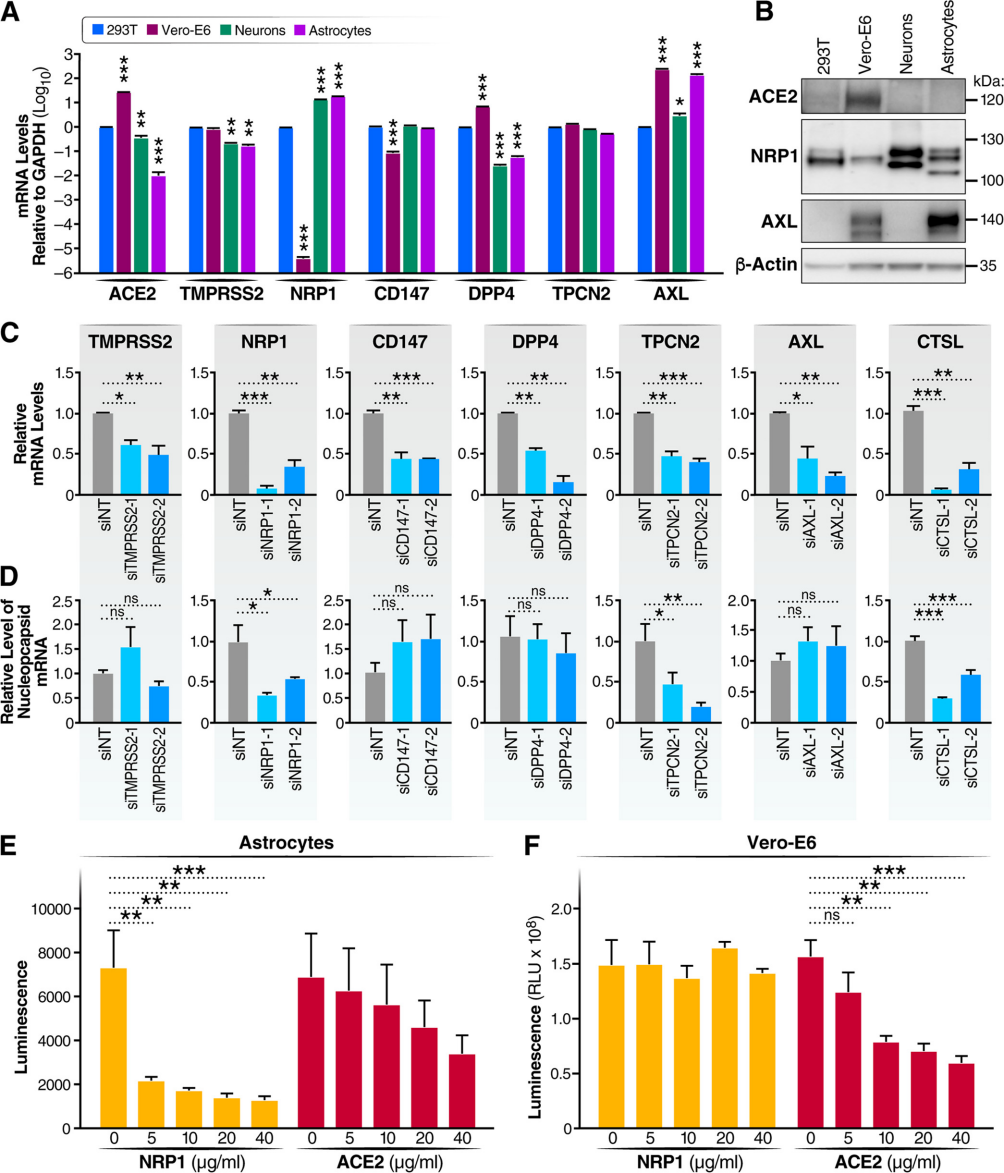
NRP1 functions as the receptor for SARS-CoV-2 in astrocytes, while TPCN2 regulates endocytosis required for entry and cleavage by cathepsin L, triggering fusion. (A) The levels of expression of several potential viral receptors were measured by RT-qPCR in 293T cells, Vero-E6 cells, astrocytes, and neurons. The results presented are from three independent experiments performed in triplicate. Error bars indicate SEM. *, *P* < 0.05; **, *P* < 0.01; ***, *P* < 0.001 (by Student’s two-tailed *t* test). (B) ACE2, NRP1, and AXL were measured by immunoblotting with specific antibodies in 293T cells, Vero-E6 cells, astrocytes, and neurons. (C and D) Two different siRNAs for each selected potential entry factor were transfected into astrocytes, and 48 h later, half of the cells were infected by SARS-CoV-2 and the other half continued in culture to determine the level of target gene knockdown. (C) Total RNAs were extracted from these cells at 3 dpi and analyzed by RT-qPCR to assess the efficiency of the knockdown of each target gene normalized to the results obtained with the siNT (nontargeting) control. (D) Viral infection was analyzed at 3 dpi by RT-qPCR and normalized to the value for the siNT group. ns, not significant; *, *P* < 0.05; **, *P* < 0.01; ***, *P* < 0.001 (data were analyzed using one-way ANOVA). Results were based on data from three independent experiments, each performed in triplicate. Data are presented as means ± SEM. (E) Astrocytes were pretreated with anti-ACE2 or anti-NRP1 antibodies, followed by SARS-CoV-2-Nluc infection. After 48 h, cells were harvested, and luciferase activity was measured. *, *P* < 0.05; **, *P* < 0.01; ***, *P* < 0.001; ns, not significant. Data were analyzed using one-way ANOVA. Representative results from one of three independent experiments performed in triplicate are shown. (F) Vero-E6 cells were pretreated with anti-ACE2 or anti-NRP1 antibodies, followed by SARS-CoV-2-Nluc infection. After 24 h, cells were harvested, and luciferase activity was measured. *, *P* < 0.05; **, *P* < 0.01; ***, *P <* 0.001; ns, not significant. Data were analyzed using one-way ANOVA. Results from a representative experiment from three independent experiments performed in triplicate are presented.

In many cells, the human angiotensin-converting enzyme 2 (ACE2) protein functions as the key receptor for SARS-CoV-2 promoting cellular entry (31). However, ACE2 is only weakly expressed in the developing or mature human cortex (28, 32, 33). Recent studies have described additional host factors that could function as entry receptors or cofactors in neural tissues (see Table S1 in the supplemental material). These proteins include neuropilin-1 (NRP1), tyrosine-protein kinase receptor UFO (AXL), basigin (CD147), dipeptidyl-peptidase 4 (DPP4), transmembrane serine protease 2 (TMPRSS2), and two-pore segment channel 2 (TPC2) (34–40). The expression of these host factors was assessed at the protein (immunoblotting) and RNA (RT-qPCR) levels in astrocytes, neurons, 293T cells, and Vero-E6 cells (Fig. 2A and B). As expected, the ACE2 protein was highly expressed in Vero-E6 cells but only weakly detected in astrocytes, suggesting the potential involvement of an alternative receptor in astrocytes. In contrast, NRP1, a recently identified receptor for SARS-CoV-2 (34, 35), was highly expressed in astrocytes and neurons but only minimally expressed in Vero-E6 cells. AXL, a second potential receptor for SARS-CoV-2, was highly expressed in Vero-E6 cells and astrocytes but not neurons (36). TMPRSS2, CD147, and TPCN2 were expressed at similar levels within each of the examined cell types. At the RNA level, the levels of NRP1 and AXL mRNAs were 140,000-fold and 200,000-fold higher, respectively, than the levels of ACE2 mRNA in astrocytes (Fig. S1A). The levels of NRP1 and AXL mRNAs were 3,000-fold and 150-fold higher, respectively, than the levels of ACE2 mRNA in neurons (Fig. S1B). These results stimulated our interest in NRP1 and AXL as potential receptors for SARS-CoV-2 entry into astrocytes.

10.1128/mbio.02308-22.1 FIG S1 (A) Relative mRNA expression levels of potential viral receptors in astrocytes normalized to ACE2 expression levels. Error bars indicate SEM for triplicate samples. This experiment was performed three times, with similar results. (B) Relative mRNA expression levels of potential viral receptors in neurons normalized to ACE2 expression levels. Error bars indicate SEM for triplicate samples. This experiment was performed three times, with similar results. (C) Astrocytes were pretreated with escalating doses of anti-ACE2 or anti-NRP1 antibodies, followed by infection with SARS-CoV-2. Seventy-two hours after infection, cell viability in the cultures was assessed using the CellTiter-Glo assay. Representative results from one of three independent experiments are shown. Error bars indicate SEM for triplicate samples. (D) Chemical inhibitors of TPCN2 (tetrandrine) or NRP1 (EG00229 and EG01377) produce dose-related inhibition of SARS-CoV-2 infection in astrocytes. Representative results from one of two independent experiments are shown. Error bars indicate SEM for triplicate samples. (E) Astrocytes were treated with various concentrations of tetrandrine, EG00229, or EG01377. Seventy-two hours later, cell viability was assessed using the Cell Titer-Glo assay. Results reflect the results obtained from three independent experiments, each performed in triplicate. Error bars indicate SEM. Download FIG S1, TIF file, 1.7 MB. Copyright © 2022 Kong et al. 2022 Kong et al. https://creativecommons.org/licenses/by/4.0/ This content is distributed under the terms of the Creative Commons Attribution 4.0 International license.

10.1128/mbio.02308-22.7 TABLE S1 Role of host factors in SARS-CoV-2 infection. Download Table S1, DOCX file, 0.05 MB. Copyright © 2022 Kong et al. 2022 Kong et al. https://creativecommons.org/licenses/by/4.0/ This content is distributed under the terms of the Creative Commons Attribution 4.0 International license.

To assess the role of NRP1 or AXL in SARS-CoV-2 infection, each was depleted in astrocytes by the introduction of sequence-specific small interfering RNAs (siRNAs), with the inclusion of a nontargeting siRNA (siNT) as a negative control. Other entry factor candidates were similarly analyzed in parallel. The siRNA knockdown efficiency was confirmed by RT-qPCR (Fig. 2C). siRNAs specific for NRP1, but not AXL, strongly inhibited the infection of astrocytes by SARS-CoV-2, as assessed by the reduced expression of viral nucleocapsid (N) mRNA (Fig. 2D). The effective knockdown of TPCN2, TMPRSS2, DPP4, and CD147 was similarly achieved (Fig. 2C), but only TPCN2 knockdown inhibited SARS-CoV-2 infection in astrocytes (Fig. 2D). TPCN2 has been reported to facilitate both SARS-CoV-2 (40) and Ebola virus (41) entry, likely by promoting the endosome movement required for the entry of both of these viruses. Further supporting a requirement for endocytosis, the knockdown of cathepsin L (CTSL) also inhibited infection (Fig. 2D). CTSL resides in the lysosome/late endosome and cleaves S2, priming this subunit for virion fusion.

The effects of the knockdown of ACE2 could not be reliably assessed due to its low baseline expression levels in astrocytes. However. treatment of primary astrocytes with a neutralizing anti-ACE2 antibody did not significantly impair SARS-CoV-2 infection, while treatment with anti-NRP1 antibody strongly and significantly blocked infection (Fig. 2E and Fig. S1C). On the basis of luminescence fold changes, we notice a trend toward a reduction in viral infection with anti-ACE2 treatment in astrocytes, which suggested that ACE2 may contribute to viral infection in astrocytes. In contrast, the anti-ACE2 antibodies significantly blocked SARS-CoV-2 infection of Vero-E6 cells, while anti-NRP1 antibodies did not (Fig. 2F). These findings support NRP1, not ACE2, as the primary receptor for SARS-CoV-2 in astrocytes.

NRP1 serves as a cell surface coreceptor for a number of different growth factors, including different isoforms of vascular endothelial growth factor (VEGF). Two NRP1 chemical inhibitors, EG00229 and EG01377, block VEGF binding to the b1 domain of NRP1. EG01377 is a more potent inhibitor than EG00229 and reacts only with NRP1, while EG00229 binds to both NRP1 and NRP2 (42). Both inhibitors produced dose-related inhibition of SARS-CoV-2 infection of astrocytes, with EG01377 displaying slightly higher potency than EG00229 (Fig. S1D) At the highest dose tested, 20 μM, EG00229 produced moderate cell toxicity, which was not observed with EG10377 (Fig. S1E). These findings provide further support for the function of NRP1 as the primary SARS-CoV-2 receptor in astrocytes.

Tetrandrine, a calcium channel blocker and inhibitor of the TPCN2 gene product, also produced dose-dependent inhibition of SARS-CoV-2 infection (Fig. S1D). Fifty percent cell death was observed at the highest drug concentration (20 μM), but at lower, nontoxic doses, viral infection was inhibited in a dose-related manner (Fig. S1E). These results provide further support implicating a role for TPCN2 in the SARS-CoV-2 entry process in astrocytes.

### Delta and Omicron variants exhibit compromised infectivity in astrocytes and brain organoids.

**FIG 3:**
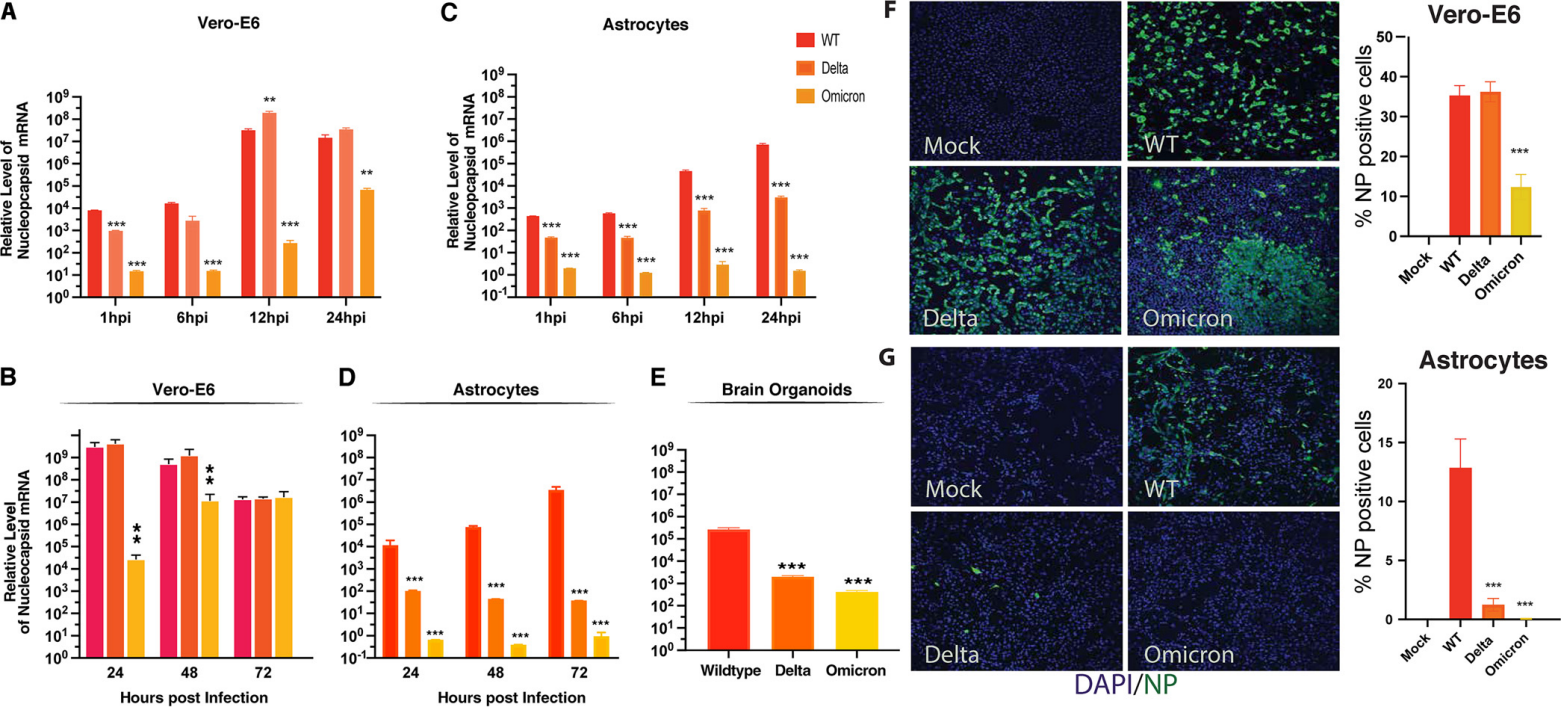
Delta and Omicron variants of concern display less efficient replication in astrocytes and brain organoids. (A) Vero-E6 cells were infected with the indicated viruses at a multiplicity of infection (MOI) of 0.01. Cell lysates were collected 1, 6, 12, and 24 h after infection. The expression of nucleocapsid viral RNA was measured by RT-qPCR and normalized based on the mock infection control. *, *P* < 0.05; **, *P* < 0.01; ***, *P* < 0.001 (by Student’s two-tailed *t* test). (B) Vero-E6 cells were infected with the indicated viruses at an MOI of 0.01. Cell lysates were collected 24, 48, and 72 h after infection. The expression of nucleocapsid viral RNA was measured by RT-qPCR and normalized to the mock infection control. *, *P* < 0.05; **, *P* < 0.01; ***, *P* < 0.001 (by Student’s two-tailed *t* test). (C) Astrocytes were infected with the indicated viruses at an MOI of 0.01. Cell lysates were collected at 1, 6, 12, and 24 h postinfection. The expression of nucleocapsid viral RNA was measured by RT-qPCR and normalized to the mock infection control. *, *P* < 0.05; **, *P* < 0.01; ***, *P* < 0.001 (by Student’s two-tailed *t* test). (D) Astrocytes were infected with the indicated viruses at an MOI of 0.01. Cell lysates were collected at 24, 48, and 72 dpi. The expression of nucleocapsid viral RNA was measured by RT-qPCR and normalized to the mock infection control. *, *P* < 0.05; **, *P* < 0.01; ***, *P* < 0.001 (by Student’s two-tailed *t* test). (E) four-month-old brain organoids were infected with the indicated viruses at an MOI of 1. After 6 h of incubation, the medium was replaced. Cell lysates were collected at 7 days postinfection. The expression of nucleocapsid viral RNA was measured by RT-qPCR and normalized to the mock infection control. *, *P* < 0.05; **, *P* < 0.01; ***, *P* < 0.001 (by Student’s two-tailed *t* test). (F) Vero-E6 cells were infected with the indicated viruses at an MOI of 0.1. Cells were fixed and immunostained at 48 h postinfection. Four image fields per group from two independent experiments were quantitated for the presence of NP. *, *P* < 0.05; **, *P* < 0.01; ***, *P* < 0.001 (by Student’s two-tailed *t* test). (G) Astrocytes were infected with the indicated viruses at an MOI of 0.1. Cells were fixed and immunostained at 48 h postinfection. Four image fields per group from two independent experiments were quantitated for the presence of NP. *, *P* < 0.05; **, *P* < 0.01; ***, *P* < 0.001 (by Student’s two-tailed *t* test).

A number of variants of concern have arisen since the inception of the pandemic. The Delta variant was estimated to be at least twice as transmissible as the original Wuhan SARS-CoV-2 (“wild type” [WT]) (43), and Delta dominantly replaced all other variants circulating as the predominant global variant. The increased transmission of the Delta variant in part involves the tighter binding of its spike to ACE2 (44). Recently, the Omicron variant has supplanted Delta in large part because of its immunoevasive properties (45). Since the ACE2 expression level is extremely low in the CNS, and NRP1 is utilized as the major receptor in astrocytes, we explored the possibility that improved ACE2 binding by the Delta variant or the introduction of numerous mutations in Omicron spike conferring immunoevasive properties might compromise binding to NRP1. To test this hypothesis, astrocytes were challenged with WT, Delta, and Omicron viruses at an MOI of 0.01. Viral RNA was measured by RT-qPCR at different time points. At early time points, in Vero-E6 cells, Delta virus showed high infectivity, exceeding that of the WT at 12 hpi. In contrast, Omicron was markedly less infectious than the other two viruses at all of the time points (Fig. 3A). Vero cells were similarly infected at 72 h by all three viruses, although Omicron lagged behind the other two viruses at 24 and 48 h (Fig. 3B).

In contrast, both the Delta and Omicron variants were significantly less infectious than the wild-type virus in astrocytes at all time points (Fig. 3C and D). When 4-month-old brain organoids were infected with the WT, Delta, or Omicron virus (MOI = 1) and viral RNA was measured at 7 dpi, the Delta and Omicron variants proved significantly less infectious than the WT virus (Fig. 3E). Additionally, immunostaining revealed that NP-positive cells in Vero-E6 cells at 48 hpi were much more abundant in WT and Delta than in Omicron infection (Fig. 3F). In contrast, in the astrocyte cultures, markedly fewer NP-positive cells were observed following Delta and Omicron infection than following WT infection (Fig. 3G).

These results revealed that the Delta is less infectious in astrocytes, which may be related to the affinity of binding between spike and NRP1. In the case of Omicron, the large number of mutations present in this variant’s spike may have compromised its ability to infect astrocytes via NRP1.

### Transcriptional and epigenetic dysregulation following SARS-CoV-2 infection of brain organoids.

**FIG 4:**
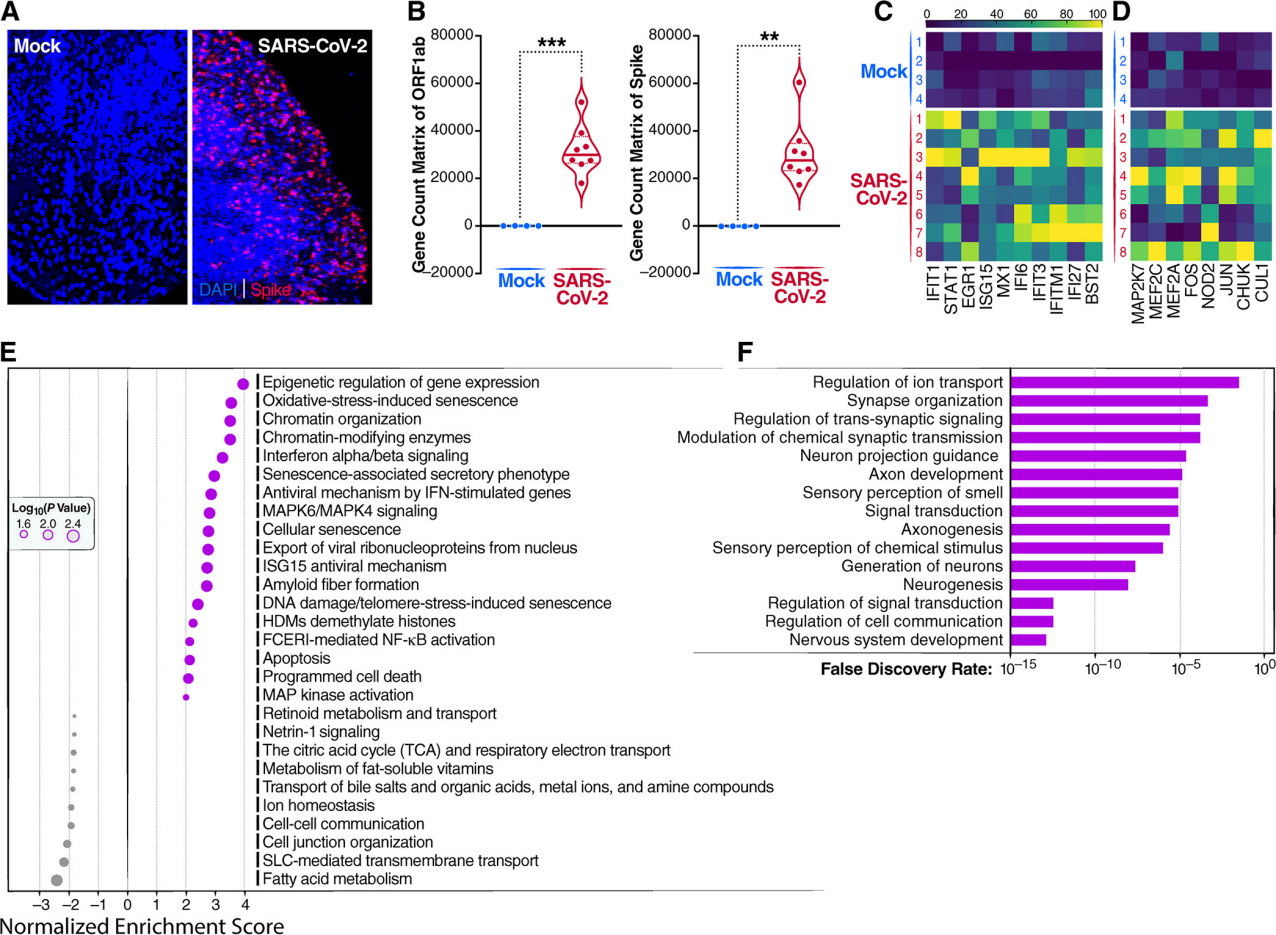
Spatial transcriptomic analysis and epigenetic analysis of SARS-CoV-2 infection of brain organoids. (A) Four-month-old cerebral organoids were either mock infected or infected with SARS-CoV-2 (WA1) at an MOI of 1 for 7 days. After 6 h of incubation, the medium was replaced. Immunofluorescence staining was performed to detect SARS-CoV spike-positive cells. Representative images are shown. (B) Expression of ORF1ab and spike detected in SARS-CoV-2-infected brain organoids. *, *P* < 0.05; **, *P* < 0.01; ***, *P* < 0.001 (by Student’s two-tailed *t* test). (C) Heat map depicting the upregulation of 10 different type I interferon-inducible genes following SARS-CoV-2 infection compared to mock-infected brain organoids. Values are shown for each replicate as Z-scores. The Z-score is the number of standard deviations a given data point lies above or below the mean. (D) Heat map of MAPK activation-related genes upregulated during SARS-CoV-2 infection relative to mock-infected brain organoids. (E) Bubble plots showing the biological processes corresponding to significantly upregulated and downregulated genes in brain organoids HDMs, histone demethylases; FCERI, Fc epsilon receptor I. (F) Brain organoids that were 110 days old were infected with SARS-CoV-2 at an MOI of 1 for 7 days. DNA was extracted from both mock- and SARS-CoV-2-infected brain organoids. Significant gene pathways enriched for DNA hypermethylation following SARS-CoV-2 infection of brain organoids are shown.

To examine gene expression changes induced by SARS-CoV-2 infection of brain organoids, NanoString GeoMx digital spatial profiling (DSP) was performed. For each NanoString slide, 12 regions of interest (ROIs) were selected based on the number of cells and the presence of spike-positive cells (Fig. 4A). NanoString DSP sequencing confirmed the presence of transcripts for open reading frame 1ab (ORF1ab) and spike in the infected brain organoids (Fig. 4B). Hierarchical clustering revealed that SARS-CoV-2 induced increased transcription of inflammatory chemokines, including CCL3 and CXCL3 (Fig. S2A and B), indicating the activation of an intrinsic inflammatory response. Type I interferon (IFN-I)-related genes, including IFIT1, IFIT3, STAT1, EGR1, ISG15, MX1, IFI6, IFI27, IFITM1, and BST2, were also strongly induced following SARS-CoV-2 infection (Fig. 4C). Pathway analysis revealed the activation of genes involved in chromatin modification (CHD1, H2BC5, H2AC12, and INO80) as well as genes mediating cell activation and apoptosis (Fig. 4E). NOD2, a member of the Nod-like receptor (NLR) family of intracellular sensors of pathogen/microbe-associated molecular patterns, was upregulated, promoting NF-κB and mitogen-activated protein kinase (MAPK) activation, which in turn stimulated the production of proinflammatory cytokines and chemokines (Fig. 4D). NOD2 sensing of SARS-CoV-2 likely plays a key role in coordinately inducing both the observed proinflammatory and innate immune responses (46).

10.1128/mbio.02308-22.2 FIG S2 (A) Four-month-old brain organoids were infected with SARS-CoV-2 at an MOI of 1 for 7 days. Hierarchical clustering was performed to compare the expression levels of genes following mock or SARS-CoV-2 infection. (B) Four-month-old brain organoids were infected with SARS-CoV-2 at an MOI of 1 for 7 days. The heat map compares the expression levels of the CCL3 and CXCL3 chemokine genes in brain organoids following mock or SARS-CoV-2 infection. (C) Four-month-old brain organoids were infected with SARS-CoV-2 at an MOI of 1 for 7 days. The heat map compares the expression levels of SLC-related genes and synapse-related genes in mock- versus SARS-CoV-2-infected brain organoids. (D) Four-month-old brain organoids were challenged with the SARS-CoV-2 at an MOI of 1 for 7 days (*n* = 6 organoids under each condition). The expression levels of nucleocapsid viral RNA and the indicated genes were measured by RT-qPCR and normalized to the mock control. *, *P* < 0.05; **, *P* < 0.01; ***, *P* < 0.001 (as determined using Student’s *t* test). (E) Four-month-old brain organoids were challenged with the indicated SARS-CoV-2 variants at an MOI of 1 for 7 days (*n* = 4 organoids under each condition). The expression levels of nucleocapsid viral RNA and the indicated genes were measured by RT-qPCR and normalized to the mock control. *, *P* < 0.05; **, *P* < 0.01; ***, *P* < 0.001 (as determined using Student’s *t* test). Download FIG S2, TIF file, 2.1 MB. Copyright © 2022 Kong et al. 2022 Kong et al. https://creativecommons.org/licenses/by/4.0/ This content is distributed under the terms of the Creative Commons Attribution 4.0 International license.

Several different classes of genes were downregulated after SARS-CoV-2 infection, including those involved in cell-cell communication, cell junction organization, ion and fatty acid homeostasis, and solute carrier (SLC)-mediated transmembrane transport (Fig. 4E). SLC-mediated transporters for thyroid hormone, ions, amino acids, choline, and energetic substrates (47) (e.g., SLC16A2, SLC41A3, SLC16A10, SLC44A1, and SLC35C2) were downregulated following viral infection (Fig. S2C). The decreased expression of these genes would likely lead to marked metabolic derangements. Lower expression levels of genes associated with synapse function, including CAMK2D, ERBB2, C1QL, and SYPL1, were also observed (Fig. S2C), suggesting that SARS-CoV-2 infection can negatively impact neuronal function. In this regard, DNA methylation profiling of SARS-CoV-2-infected brain organoids also revealed the hypermethylation of numerous genes involved in neurogenesis, axonogenesis, and synaptic transmission (Fig. 4F and Fig. S3 and S4).

10.1128/mbio.02308-22.3 FIG S3 Brain organoids that were 110 days old were infected with SARS-CoV-2 at an MOI of 1 for 7 days. DNA was extracted from both mock- and SARS-CoV-2-infected brain organoids. The heat map depicts changes in the DNA hypermethylation of genes involved in neurogenesis, synapse organization, and the regulation of signal transduction in brain organoids following mock or SARS-CoV-2 infection. Download FIG S3, TIF file, 2.4 MB. Copyright © 2022 Kong et al. 2022 Kong et al. https://creativecommons.org/licenses/by/4.0/ This content is distributed under the terms of the Creative Commons Attribution 4.0 International license.

10.1128/mbio.02308-22.4 FIG S4 Brain organoids that were 110 days old were infected with SARS-CoV-2 at an MOI of 1 for 7 days. DNA was extracted from both mock- and SARS-CoV-2-infected brain organoids. The heat map depicts changes in the DNA hypermethylation of genes involved in axonogenesis, modulation of chemical synaptic transmission, and nervous system development in brain organoids following mock or SARS-CoV-2 infection. Download FIG S4, TIF file, 2.4 MB. Copyright © 2022 Kong et al. 2022 Kong et al. https://creativecommons.org/licenses/by/4.0/ This content is distributed under the terms of the Creative Commons Attribution 4.0 International license.

RT-qPCR was used to validate the activation of several genes in the type I interferon pathway, including MX1, IFI6, and ISG15 (Fig. S2D). The proinflammatory chemokines CCL3, CXCL3, and CXCL10 were also upregulated following infection, while synapse-related genes, including C1QL1 and SYPL1, were downregulated (Fig. S2D). Of note, the expression of the major water channel in astrocytes, aquaporin-4 (AQP4), was strikingly inhibited following viral infection. Together, these results suggest that SARS-CoV-2 triggers a potentially pathological interferon response that combines with an inflammatory response to disrupt energy homeostasis and the transport of water, nutrients, and neurotransmitters. These events culminate in a microenvironment that promotes neuronal dysfunction. Of note, the Delta and Omicron variants, although less infectious in the brain organoids, also induced similar levels of interferon activation, inflammation, and downregulation of AQP4 (Fig. S2E), arguing for retention of neurotoxic potential.

### Transcriptional and epigenetic changes following SARS-CoV-2 infection of primary astrocytes.

**FIG 5:**
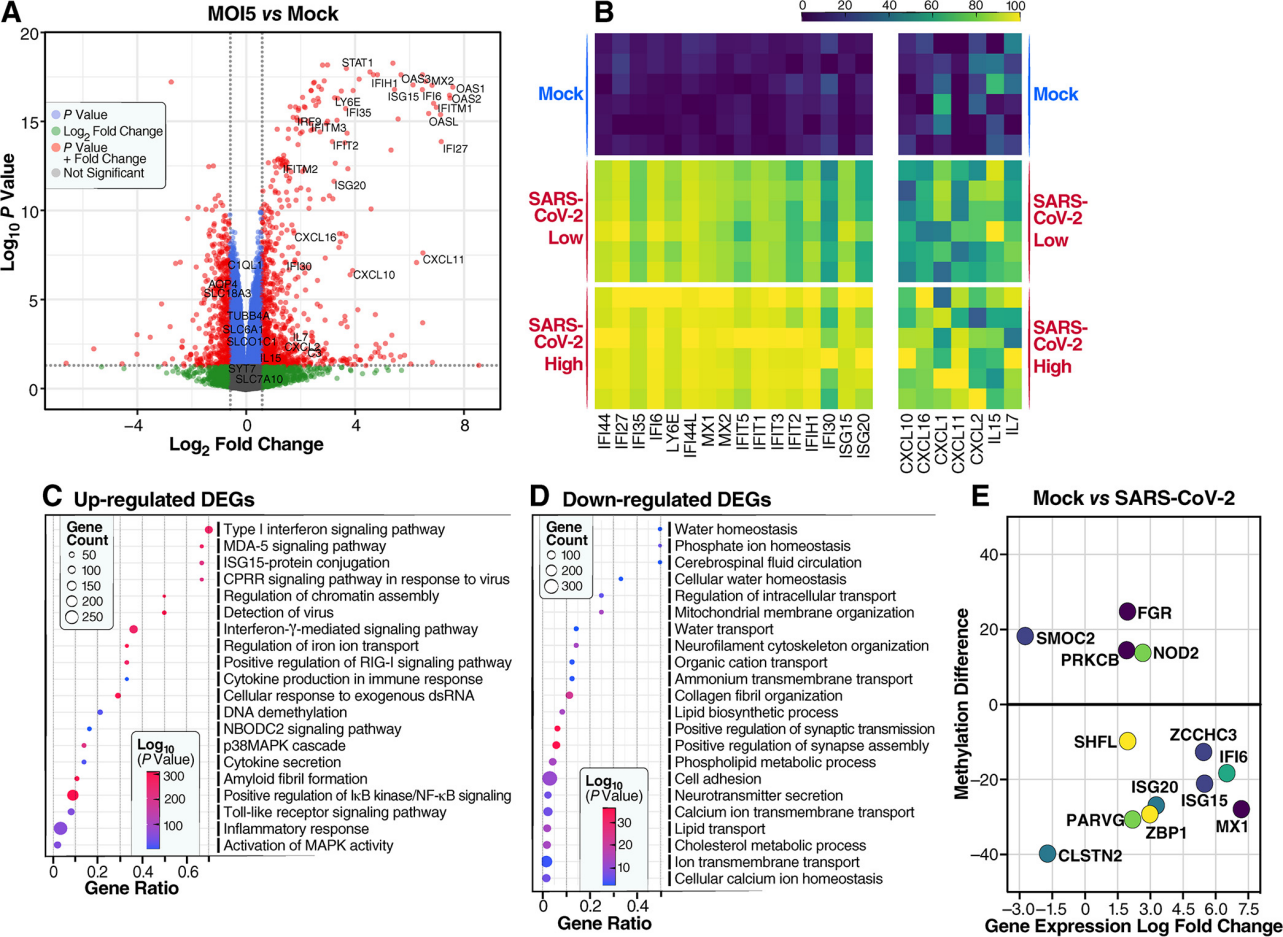
SARS-CoV-2 infection induces marked transcriptional and epigenetic changes in two-dimensional cultures of primary astrocytes. (A) Volcano plot of changes in gene expression comparing mock-infected to SARS-CoV-2 (WA1)-infected cultures (MOI = 5) at 96 hpi. Selected changes in type I IFN response genes, inflammatory genes, and genes involved in ion transport are highlighted. (B) Heat map of interferon-inducible and inflammation-related genes upregulated during SARS-CoV-2 infection of primary astrocytes. Astrocytes were infected by SARS-CoV-2 at an MOI of 1 (SARS-CoV-2 low) or an MOI of 5 (SARS-CoV-2 high). (C) Bubble plots showing the significant biological processes of upregulated differentially expressed genes (DEGs) in astrocytes. CPRR, cytoplasmic pattern recognition receptor; dsRNA, double-stranded RNA. (D) Bubble plots showing the significant biological processes of downregulated DEGs in infected astrocytes. (E) DNA methylation differences in DEGs following mock or SARS-CoV-2 infection at an MOI of 5 at 96 hpi. *SMOC2* (intron), *FGR* (exon), *PRKCB* (intron), *NOD2* (intron), *SHFL* (promoter), *ZCCHC3* (promoter), *IFI6* (intron), *ISG15* (intron), *ISG20* (promoter), *ZBP1* (exon), *MX1* (promoter), *PARVG* (intron), and *CLSTN2* (intron) are shown.

To further explore these changes in host gene expression, we used RNA-seq to assess the effects of SARS-CoV-2 infection at an MOI of 1 or 5 in two-dimensional (2D) cultures of primary human astrocytes (Fig. 5 and Fig. S5). A marked upregulation of type I IFN-stimulated genes (ISGs), including IFI144, IFI27, IFI35, IFI6, IFI30, IFI44L, LY6E, MX1, MX2, IFIT1, IFIT2, IFIT3, IFIT5, ISG20, and ISG15, was observed in infected cells (Fig. 5A and B). These findings are consistent with the results obtained in the brain organoids. The upregulation of various IFN-induced oligoadenylate synthetase (OAS) antiviral genes, including OAS1, OAS2, OAS3, and OASL, was also detected in the infected astrocytes (Fig. S5E), further confirming the activation of type I IFN signaling in astrocytes. SARS-CoV-2-infected astrocytes also exhibited increased transcription levels of many inflammatory chemokines/cytokines, including CXCL10, CXCL16, CXCL1, CXCL2, interleukin-15 (IL-15), and IL-7 (Fig. 5B). Gene ontology (GO) and WikiPathways analyses of the upregulated genes in infected cells revealed the enrichment of genes induced by type I, II, and III interferons and genes involved in Toll-like receptor (TLR), MDA5, and RIG-I sensing of double-stranded RNA. Additionally, the levels of genes involved in DNA demethylation, proinflammatory responses, apoptosis, p38 MAPK signaling, and neurodegenerative signaling like that found in Parkinson’s disease and amyotrophic lateral sclerosis were also increased (Fig. 5C and Fig. S5F). Overall, these results obtained in the 2D cultures of astrocytes were in agreement with the results found in the brain organoids and suggest a key role for NOD2 in the induction of both the interferon and inflammatory pathways (Fig. S5E).

10.1128/mbio.02308-22.5 FIG S5 Studies of primary human astrocytes. (A) Principal-component analysis of sample relationships. Each point represents an RNA-seq sample. Samples with similar gene expression profiles are clustered. (B) Levels of potential receptor RNA expression in mock- or SARS-CoV-2-infected astrocytes. Astrocytes were infected by SARS-CoV-2 at an MOI of 1 (SARS-CoV-2 low) or an MOI of 5 (SARS-CoV-2 high). (C) Changes in SARS-CoV-2 RNA expression following infection of astrocytes for 96 h at an MOI of 1 (low) or an MOI of 5 (high). (D) Volcano plot of gene expression changes 96 h after mock or SARS-CoV-2 infection at an MOI of 1. Changes in the expression levels of several IFN response genes, genes involved in inflammation, and ion transport genes are highlighted. (E) Heat map demonstrating the upregulation of genes in different signaling pathways following mock versus SARS-CoV-2 infection for 96 h. (F) Bubble plots depicting the upregulation of various pathways as indicated by the upregulated DEGs in astrocytes. (G) Heat map of selected downregulated genes in SARS-CoV-2-infected versus mock-infected astrocytes measured 96 h after infection. Download FIG S5, TIF file, 2.9 MB. Copyright © 2022 Kong et al. 2022 Kong et al. https://creativecommons.org/licenses/by/4.0/ This content is distributed under the terms of the Creative Commons Attribution 4.0 International license.

The activation-induced deaminase (AID)/APOBEC family cytosine deaminases, known to promote the affinity maturation of antibodies and to function as host restriction factors for various viruses, also promote DNA demethylation leading to transcriptional activation (48).

Specifically, these enzymes deaminate 5-methylcytosine (5mC) or 5-hydroxymethylcytosine (5hmC), thereby removing the target of DNA methylation. SARS-CoV-2 infection of astrocytes upregulates the expression of APOBEC3B, APOBEC3C, APOBEC3D, APOBEC3G, and APOBEC3F. Using DNA methylation profiling, we identified 2,596 differentially methylated sites where methylation changed either up or down by 10% or more following SARS-CoV-2 infection of astrocytes (MOI = 5). (false discovery rate [FDR] of <0.05) (Fig. 5E). Thirty-six percent of these sites mapped to promoter or exon regions of annotated genes. Integrative analysis with matching transcriptomic data identified 435 overlapping genes that were both differentially expressed and differentially methylated following SARS-CoV-2 infection. Of note, the MX1 gene displayed the highest log fold change in expression (>6-fold), which was accompanied by a 25% decline in promoter methylation. ISG20 and ZCCHC3 also became hypomethylated at their promoters during SARS-CoV-2 infection.

These findings suggest that aberrant gene expression programs during SARS-CoV-2 infection include a subset of genes regulated by epigenetic modification involving DNA methylation.

GO analyses of downregulated genes in infected astrocytes again revealed the enrichment of genes involved in water transport, synaptic transmission, ion and lipid transport, the formation of cell junctions, and calcium binding (Fig. 5D). These included AQP4, the predominant water channel found within the foot processes of astrocytes, and a number of SLC-mediated transporters, including SLC1A2, SLC7A10, SLC18A3, SLC6A1, SLC4A10, and SLCO1C1. These gene products are involved in the transport of many substrates, including glutamate, amino acids, vesicular acetylcholine, γ-aminobutyric acid, sodium-coupled bicarbonate, and organic anions (Fig. S5G). The downregulation of genes involved in water and solute carrier transport underscores how SARS-CoV-2 infection disrupts fundamental cellular processes, altering homeostasis within astrocytes. Additionally, the marked decrease in the expression of genes involved in synapse maintenance, including SYNPR, SYT, C1QL1, and TUBB4A (Fig. S5G), within astrocytes further highlights an indirect mechanism through which SARS-CoV-2 can adversely affect synaptic transmission and overall neuronal function.

In summary, SARS-CoV-2 infection of astrocytes triggers multiple pathways, including type I, II, and III interferon signaling; RIG-I, MDA5, and NOD2 sensing; and inflammation, while also impairing multiple pathways that directly control the health and function of neighboring neurons. Infection ultimately leads to neuronal dysfunction and death (Fig. S6).

10.1128/mbio.02308-22.6 FIG S6 SARS-CoV-2 infection induces marked transcriptional and epigenetic changes in two-dimensional cultures of primary astrocytes. An interaction map of enriched pathway terms is shown. Selected GO terms (gray sets) significantly enriched with up- and downregulated genes (red- and blue-filled circles, respectively) are connected by high-confidence interactions (score of ≥8.0) from the STRING database. The set combining type I, II, and III interferon signaling genes is highlighted in yellow. Type III pathway genes are from WikiPathways, substituting the corresponding GO term, which was empty at the time of this study. The thickness of the connection scales with the number of interactions from 1 to 40. The node fill color is based on a gradient of log_2_ fold changes in values ranging from −3 (blue) to 3 (red). The thick red node border indicates significant differential expression (*P* < 0.05) for an individual gene. CPRR signaling, cytoplasmic pattern recognition receptor signaling pathway in response to virus; NBOCD2 signaling, nucleotide binding oligomerization domain-containing 2 signaling pathway; Ammonium TM transport, ammonium transmembrane transport. Download FIG S6, TIF file, 2.8 MB. Copyright © 2022 Kong et al. 2022 Kong et al. https://creativecommons.org/licenses/by/4.0/ This content is distributed under the terms of the Creative Commons Attribution 4.0 International license.

### SARS-CoV-2 infection induces neural cell death.

**FIG 6:**
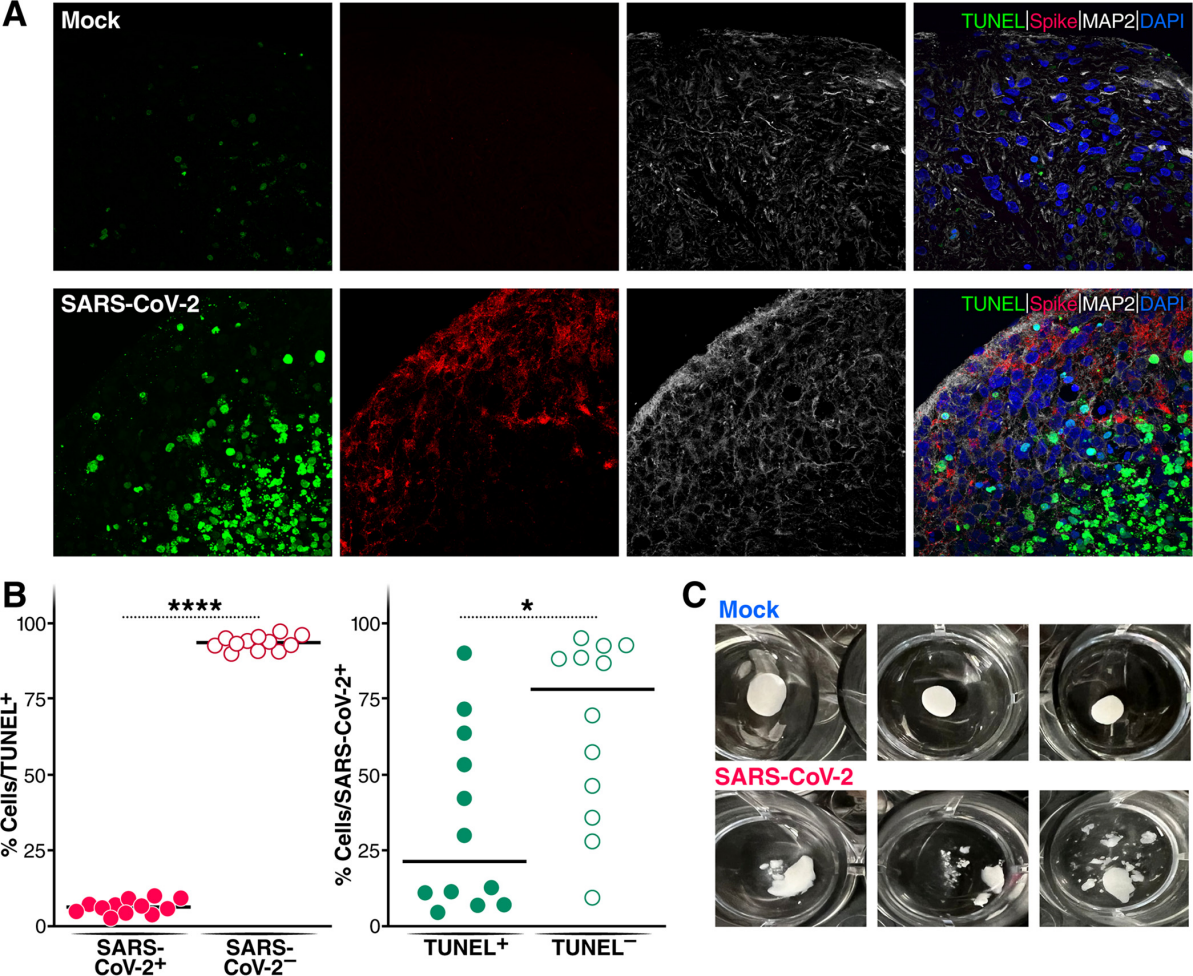
SARS-CoV-2 infects brain organoids and prominently induces bystander cell death. (A) Four-month-old cerebral organoids were either mock infected or infected with SARS-CoV-2 (WA1) for 7 days. TUNEL staining (green) was performed to evaluate cell death. Immunofluorescence staining for neurons (MAP2) (white) and spike (red) was also carried out. (B) Quantification of SARS-CoV-2 and TUNEL double-positive or SARS-CoV-2-negative, TUNEL-positive cells over the total TUNEL-positive cells. Quantification of SARS-CoV-2 and TUNEL double-positive or SARS-CoV-2-positive, TUNEL-negative cells over the total SARS-CoV-2-positive cells. All experiments were performed with images from 12 regions from two independent experiments. *, *P* < 0.05; **, *P* < 0.01; ***, *P* < 0.001; ****, *P* < 0.0001 (by Student’s two-tailed *t* test). (C) Brain organoids that were 110 days old were challenged with SARS-CoV-2 at an MOI of 1. After 12 days of infection, the structural integrity of the infected organoids sharply decreased, while the mock-infected group remained intact.

We next examined cell death following SARS-CoV-2 infection in the brain organoids. Using a terminal deoxynucleotidyltransferase-mediated dUTP-biotin nick end labeling (TUNEL) assay, we observed extensive DNA fragmentation of MAP2^+^ neurons following SARS-CoV-2 infection (Fig. 6A). Of note, the majority of these TUNEL-positive neurons were SARS-CoV-2 negative, suggesting that the neurons were not dying as a consequence of direct viral infection but rather as bystander cells (Fig. 6B). Strikingly, we observed that the brain organoids “fell apart” after 12 days of infection (Fig. 6C), suggesting that SARS-CoV-2-induced neuronal cell death undermines the structural integrity of the brain organoids. Together, these findings suggest that while SARS-CoV-2 can infect multiple CNS cells, infection of astrocytes creates a microenvironment where many bystander neurons are unable to survive.

## DISCUSSION

While COVID-19 can produce a variety of neurological deficits ranging from anosmia and dysgeusia (loss of sense of smell and taste) to encephalopathy and stroke, it is unclear whether these processes reflect direct infection of brain cells, indirect effects related to inflammation and immune cell dysfunction, or microangiopathy induced by viral infection of endothelial cells. It is possible, and indeed likely, that both direct and indirect mechanisms of neural damage occur in infected individuals. In our brain organoids, there was an abundance of astrocytes, neurons, and neural progenitor cells. Using this model, we find that astrocytes support high levels of SARS-CoV-2 infection, with lower levels of infection occurring in neurons and neural progenitor cells. The results of three recent research studies employing brain organoids and postmortem human brain tissue support our findings (15, 25, 28). However, one study revealed that SARS-CoV-2 preferentially infects neurons in brain organoids. Of note, the proportion of astrocytes in their model was low due to the use of “young” brain organoids (16). Our findings suggested that targeting NRP1 or TPCN2 with chemicals was a potential therapeutic strategy for COVID-19.

In most tissues, ACE2 functions as the major cell entry receptor for SARS-CoV-2. However, ACE2 levels are quite low in the CNS, including in astrocytes. While alternative receptors, including NRP1, TPCN2, CD147, NRP1, and AXL, have been proposed to mediate SARS-CoV-2 infection in various cell types (see Table S1 in the supplemental material), no systematic studies have evaluated what receptor mediates SARS-CoV-2 entry into astrocytes. Using siRNAs to knock down the expression of these individual receptors, we find that NRP1 and TPCN2 are required for SARS-CoV-2 entry into and productive infection of astrocytes.

However, contrasting results have also been described. One recent study suggests that ACE2 receptors can mediate SARS-CoV-2 infection of both neurons and astrocytes but that these infections are specifically confined to the choroid plexus and paraventricular nuclei of the thalamus where ACE2 receptor expression is elevated (49). A second study suggests that SARS-CoV-2 infection of neurons occurs via ACE2 receptors but culminates in an abortive, nonspreading form of infection (50). This result differs sharply from the productive infection that we observe in astrocytes following the NRP1-dependent entry of SARS-CoV-2. These findings likely highlight the intrinsic experimental challenges posed by the biological and spatial complexity of the brain.

NRP1 corresponds to a single-pass transmembrane protein that regulates both CNS and cardiovascular development. NRP1 naturally functions as a receptor for semaphorin 3A mediating growth cone guidance but also binds the VEGF-165 isoform of VEGF-A promoting vascular development. Of note, NRP1 is known to bind furin-cleaved substrates (34, 51), and the SARS-CoV-2 spike protein contains a furin cleavage site that is proteolytically cleaved to separate the S1 receptor binding subunit from the S2 subunit mediating fusion. Furin cleavage, which often occurs as the virion buds from the producer cell, likely creates the ligand for the engagement of NRP1.

The TPCN2 gene encodes the TPC2 protein, a two-pore calcium channel that regulates endocytosis. The knockdown of TPCN2 also inhibits Ebola virus infection by blocking the endocytosis required for infection (41). We suspect that TPCN2 functions similarly in the context of SARS-CoV-2 infection of astrocytes, with fusion occurring in late endosomes after endocytosis.

### Delta and Omicron variants of SARS-CoV-2 exhibit lower infectivity in astrocytes and brain organoids.

One of the key features of RNA viruses is their ability to adapt over time as they attempt to improve transmission and growth within their hosts. Most changes have little to no impact on the virus’s life cycle, but some may affect its transmission or virulence or even the effectiveness of vaccine countermeasures. The Delta variant (B.1.617.2) arose in December 2020 during the surge of infections in India and subsequently spread to over 160 countries, establishing itself as the dominant global strain for many months prior to the emergence of the Omicron variant. Delta infectivity is increased approximately 2-fold over that of the ancestral Wuhan-Hu-1 strain, likely due to the higher-affinity binding of S1 to ACE2 receptors and the more rapid kinetics of S2-mediated fusion (44, 52). The high infection rate of the Delta variant is attributed to several mutations in its spike protein, including T19R, G142D, Δ156–157, R158G, L452R, T478K, D614G, P681R, and D950N. Mutations outside spike, for example, in the nucleocapsid protein of Delta (R203M), also contribute to increased viral spread (53). The combination of these mutations leads to shorter incubation times and markedly higher nasopharyngeal viral loads, favoring more effective spread. The Omicron variant contains more than 30 mutations distributed in spike and other genes. A recent study showed that the Omicron variant is attenuated *in vitro* and *in vivo* compared to the WT and other variants (54), arguing that its immunoevasive properties importantly contribute to its rapid spread.

We were interested in whether the changes in Delta’s spike that improved entry via ACE2 receptors might compromise the ability of this spike to utilize the NRP1 receptor. Indeed, we find a less effective spread of Delta viruses in astrocyte cultures and brain organoids, where NRP1 functions as the receptor, than the WT strain. In contrast, Delta replicates similarly to the WT in Vero-E6 cells, where ACE2 functions as the principal receptor. This apparent trade-off in the efficiency of receptor use is noteworthy as this process could lead to variants displaying different pathological effects within different target tissues or organs. Future experiments (Biacore) measuring the absorption rate constants (*K_a_*s) and dissociation constants (*K_d_*s) for the interaction of different spike proteins with ACE2 versus NRP1 will better define the molecular basis of these apparent differences in receptor utilization.

### SARS-CoV-2 infection and interferon induction in astrocytes and brain organoids.

Interferons (IFNs) are innate and adaptive cytokines involved in many biological responses, particularly the host response to viral infections. While the early upregulation of type I IFN signaling may protect host cells from the virus, sustained activation coupled with the long-term expression of interferon-stimulated genes is linked to several CNS diseases characterized as “interferonopathies” (55). This process likely occurs during COVID-19 infection in the CNS. An early protective role is supported by the fact that loss-of-function mutations in the TLR7 gene mediating IFN-I induction or the development of autoantibodies against type I IFNs are linked to the development of severe COVID-19 (56, 57). Conversely, the heightened and prolonged production of IFNs in patients infected with SARS-CoV-2 is correlated with negative clinical outcomes (58, 59). Our studies reveal that SARS-CoV-2 infection leads to marked inductions of type I interferon production in brain organoids and type I, II, and III interferon production in primary astrocytes. In contrast, Song and colleagues reported that SARS-CoV-2 infection does not upregulate type I interferon in brain organoids (16). The reason for this discrepancy is unclear, but Song and coworkers used young brain organoids that likely contained few astrocytes, and their comparisons were limited largely to infected neurons and bystander neurons. In contrast, we have used brain organoids at a more mature stage that contained large numbers of astrocytes, which may explain the pronounced biological differences that we observe. The prolonged production of IFN by infected astrocytes may contribute to CNS pathology that occurs following SARS-CoV-2 infection.

### CNS inflammation driven by SARS-CoV-2 infection of astrocytes promotes neuronal dysfunction and death.

Through their intimate interactions with neurons, astrocytes play a key role in supporting neurons by modulating synapse formation and function and maintaining glutamate, ion, and water homeostasis (60). Viral infections are known to trigger reactive astrogliosis, where these cells shift to a destructive proinflammatory phenotype, accompanied by the release of various chemokines and neurotoxic factors (61). These effects create a microenvironment that promotes neuronal dysfunction and even neuronal cell death. We suggest that SARS-CoV-2 is producing such a reactive astrogliosis. The marked induction of type I interferon in infected brain organoids and astrocytes coupled with the expression of proinflammatory chemokines/cytokines, including CXCL10, CXCL3, CXCL16, CXCL1, CXCL2, CCL3, IL-15, and IL-7, is consistent with a reactive and pathological astroglial response. As a predictable consequence, we observe that bystander neuronal cell death occurs in the infected brain organoids. Reactive astrocytes are also known to display defective ion channels and impaired transporters. Indeed, we find sharp decreases in the expression of water channels (AQP4) and various SLC transporters, including transporters for glutamate (SLC1A2), neurotransmitters (SLC18A3), ions (SLC4A10, SLCO1C1, and SLC41A3), and choline (SLC44A1), in both infected brain organoids and primary astrocyte cultures. Glutamate is the predominant excitatory neurotransmitter in the mammalian CNS and is critical for excitatory synaptic transmission, synaptic plasticity, and neuronal development. Glutamate transporter 1 (SLC1A2) is responsible for the largest proportion of glutamate transport in the hippocampus and is expressed mainly in astrocytes. SLC1A2 deficiency impairs long-term potentiation (LTP) (62), a well-established form of synaptic plasticity linked to the establishment of memory. AQP4 is the major water channel in the adult brain and is expressed primarily in astrocytes. AQP4 deficiency impairs synaptic plasticity and associative fear memory in the lateral amygdala via the downregulation of SLC1A2 expression (63). Choline is vital for protecting the brain from neuropathological changes associated with Alzheimer’s disease as well as neurological damage associated with epilepsy and fetal alcohol syndrome (64). Choline transporter-like 1 (CTL1/SLC44A1) deficiency is also associated with neurodegeneration occurring during childhood (65). SLC4A10 deficiency, SLC7A10 deficiency, and variants in SLC18A3 are similarly associated with neurodegenerative disorders (66–68), while SLC16A2 and SLCO1C1 deficiency may result in blood-brain barrier dysfunction (69). Together, the panoply of genetic alterations associated with SARS-CoV-2 infection of brain organoids is most consistent with a primary effect on astrocytes leading to reactive astrogliosis that ultimately culminates in bystander neuronal cell dysfunction and death.

## MATERIALS AND METHODS

### Viruses and cells.

SARS-CoV-2/human/USA/USA-WA1/2020 (WA1) (catalog number NR-52281; BEI Resources), Delta B.1.617.2 (catalog number NR-55611; BEI Resources), and Omicron B.1.1.529 (California Department of Health) were used for cell culture infection studies. The virus infection experiments were performed in a biosafety level 3 laboratory. All of the SARS-CoV-2 strains were kindly provided to us by Melanie Ott. An infectious SARS-CoV-2 clone containing a NanoLuc reporter (SARS-CoV-2-Nluc) was kindly provided to us by Vineet Menachery (UTMB). Viral titers were determined by a 50% tissue culture infective dose (TCID_50_) assay using Vero-E6 cells (ATCC). Vero-E6 and HEK293 cells were cultured in complete Dulbecco modified Eagle medium (DMEM) consisting of 10% fetal bovine serum (FBS), penicillin (100 U/mL), and streptomycin (100 μg/mL). Human astrocytes isolated from the human brain (cerebral cortex) (catalog number 1800) were purchased from ScienCell Research Laboratories and maintained in astrocyte medium (AM) (catalog number 1801) supplemented with 10% FBS, 1% astrocyte growth supplement (AGS) (catalog number 1852), penicillin (100 U/mL), and streptomycin (100 μg/mL).

### iPSC maintenance and brain organoid differentiation.

Wild-type WTC11 line iPSCs were maintained in mTeSR plus (Stemcell Technologies) on Matrigel (8 μg/mL; Corning)-coated cell culture plates at 37°C with 5% CO_2_. Cells were passaged every 4 to 5 days using ReLeSR (Stemcell Technologies) and supplemented with Rock inhibitor Y-27632 (SelleckChem) for 24 h after each passage. Cerebral organoids were generated according to the manufacturer’s instructions for the commercial STEMdiff cerebral organoid kit (Stemcell Technologies). Human iPSC colonies were dissociated into single-cell suspensions with a gentle cell dissociation reagent (Stemcell Technologies). In total, around 9,000 cells were then plated into each well of a V-bottom ultralow-attachment 96-well plate in embryo body (EB) formation medium supplemented with 20 μM Y-27632. On day 2 and day 4, 100 μL of EB medium was added to each well without disturbing the EBs. On day 5, EBs were moved to 24-well low-attachment plates in neural induction medium for another 3 days. EBs were further embedded in 15 μL of Matrigel and cultured in neural expansion medium for 3 days in 6-well low-attachment plates to allow organoid formation. The organoids were next cultured in neural culture medium and moved to an orbital shaker for further culture. Cerebral organoids are composed of neuronal progenitors, radial glia, and neurons.

### iPSC-derived neurons.

Neuron differentiation was performed as previously described (70). For predifferentiation, i^3^N iPSCs were incubated with doxycycline (2 μg/mL) for 3 days at a density of 2.0 × 10^6^ to 2.5 × 10^6^ cells/well in six-well plates coated with Matrigel in knockout DMEM (KO-DMEM)–F-12 medium containing N2 supplement, nonessential amino acids (NEAA), laminin (0.2 μg/mL), brain-derived neurotrophic factor (BDNF) (10 ng/mL), neurotrophin-3 (NT3) (10 ng/mL; Peprotech), and Y-27632. The medium was changed daily, and Y-27632 was removed after day 2. For maturation, predifferentiated i^3^N precursor cells were dissociated, counted, and subplated at the desired density on plates coated with poly-d-lysine (PDL)–laminin in maturation medium containing 50% DMEM–F-12 medium, 50% neurobasal A medium, 0.5× B27 supplement, 0.5× N2 supplement, GlutaMAX, NEAA, laminin (1 μg/mL), BDNF (10 ng/mL), and NT3 (10 ng/mL). Half of the medium was replaced on day 7 and again on day 14, and the medium volume was doubled on day 21. Thereafter, one-third of the medium was replaced weekly until the cells were used.

### Western blotting.

Cell lysates were extracted in radioimmunoprecipitation assay (RIPA) lysis buffer (Thermo Fisher). The protein concentrations were determined by a bicinchoninic acid (BCA) assay (Thermo Fisher), and the proteins were subjected to 4 to 12% SDS-PAGE and transferred onto a polyvinylidene difluoride (PVDF) membrane. The membrane was blocked with 5% nonfat dried skim milk and incubated with the specific antibodies anti-ACE2 (catalog number AF933; R&D) (1:1,000), anti-NRP1 (catalog number MA5-32179; Thermo Fisher) (1:1,000), and anti-AXL (catalog number AF154; R&D) (1:1,000). Protein bands were detected using an enhanced chemiluminescence image analyzer. β-Actin (catalog number A5316; Millipore-Sigma) (1:5,000) was used as an internal control to assess the comparability of protein loading.

### Immunocytochemistry.

Cerebral organoids were fixed in 4% paraformaldehyde overnight at 4°C and then washed with phosphate-buffered solution (PBS) three times. After fixation, organoids were dehydrated with 30% sucrose in PBS at 4°C. Organoids were then embedded with optical cutting temperature (OCT) compound (VWR) and frozen on dry ice. Frozen tissue was sectioned at 10 μm using a cryostat and collected on ultrafrosted glass microscope slides. Sections were stored at −80°C until use. Sections were permeabilized in 0.25% Triton X-100 and blocked with blocking buffer containing 5% normal goat serum and 2% bovine serum albumin (BSA) in PBS for 2 h at room temperature (RT). Sections were then incubated with primary antibodies in blocking buffer overnight at 4°C. The primary antibodies and their dilutions used in this study are as follows: anti-MAP2 (catalog number MAB3418; Millipore-Sigma) (1:1,000), anti-TUJ1 (catalog number 801201; BioLegend) (1:1,000), anti-NESTIN (catalog number ABD69; Millipore-Sigma) (1:1,000), anti-GFAP (catalog NB300-141; Novus Biologicals) (1:1,000), antispike (catalog number GTX632604; GeneTex) (1:50), and antinucleocapsid (catalog number GTX635689; GeneTex) (1:50). After washing three times with PBS, samples were incubated with fluorescently conjugated secondary antibodies (Alexa Fluor 488, 568, and 647 conjugates; Invitrogen) (1:500) for 1 h at room temperature and washed three times with PBS before mounting a glass coverslip. Fluorescence was detected using an Olympus FV3000 confocal microscope and quantified using ImageJ software.

TUNEL imaging assays were performed according to the manufacturer’s instructions.

Astrocytes were washed with PBS and fixed in 4% paraformaldehyde for 15 min, followed by permeabilization with 0.2% Triton X-100 and blocking with 5% FBS for 1 h at RT. Antibody dilution buffer was composed of PBS supplemented with 2.5% FBS. Samples were incubated with antispike (catalog number GTX632604; GeneTex) (1:50) and antinucleocapsid (catalog number GTX635689; GeneTex) (1:50) primary antibodies overnight at 4°C, followed by three washes with PBS and incubation with fluorescence-conjugated secondary Abs at 1:500 in antibody buffer for 1 h at RT. Images were acquired using an Olympus FV3000 confocal microscope and processed using ImageJ software.

### Quantitative analysis of immunostained sections.

Quantification was conducted using CellProfiler software and ImageJ Fiji (NIH). To analyze the amount of marker expression in brain organoids, we used ImageJ Fiji software to trace and calculate the total 4′,6-diamidino-2-phenylindole (DAPI)-positive area in four immunostained sections per organoid and then determined the percentage of each marker-positive area out of the total DAPI-positive area. We used ImageJ Fiji software to calculate colocalization by quantifying the percentage of the marker-positive area over the percentage of the spike-positive area.

For TUNEL assay quantification, we used the open-source image-denoising tool Aydin (71) to denoise the nucleus channel to facilitate subsequent analysis. Next, we segmented the nuclei using the cellpose tool (72). We postprocessed nuclear labels using pyclesperanto (73). TUNEL quantities per cell were estimated by summing the pixel intensity of the TUNEL channel enclosed by each nucleus segment. The spike quantity was estimated as the mean intensity of the spike image enclosed by dilated nucleus segmentation. The threshold for TUNEL- and spike-positive cells was manually determined.

### siRNA transfection into astrocytes.

For siRNA assays, astrocytes were plated onto 24-well plates and transiently transfected with siRNA using RNAiMAX reagents. The final concentration of the siRNAs was 40 nM. Three days after transfection, cells were infected with SARS-CoV-2 at an MOI of 0.01. After 3 days of infection, cells were harvested for measurements of viral gene expression and siRNA knockdown efficiency.

### RT-qPCR.

Cells were lysed with RLT buffer (Qiagen) supplemented with 1% β-mercaptoethanol (catalog number 1610710; Bio-Rad), and total RNA was isolated using the RNeasy micro kit plus (Qiagen). Following the elution of RNA, the concentration and purity were determined using a NanoDrop 2000c spectrophotometer (Thermo Fisher Scientific). A total of 0.2 to 1 μg of RNA was reverse transcribed using the iScript cDNA synthesis kit (Bio-Rad). The real-time PCR assay was performed using the iTaq universal SYBR green supermix (Bio-Rad) and gene-specific primers (see Table S2 in the supplemental material). PCRs were performed on a Bio-Rad CFX connect qPCR instrument under the following conditions: 95°C for 10 min, 40 cycles of 95°C for 15 s, and 60°C for 1 min. The relative percentage of gene expression was normalized to the glyceraldehyde-3-phosphate dehydrogenase (GAPDH) control and was calculated using the formula 2(Δ*C_T_* of gene − Δ*C_T_* of GAPDH).

10.1128/mbio.02308-22.8 TABLE S2 Primers used for RT-qPCR in this study. Download Table S2, DOCX file, 0.02 MB. Copyright © 2022 Kong et al. 2022 Kong et al. https://creativecommons.org/licenses/by/4.0/ This content is distributed under the terms of the Creative Commons Attribution 4.0 International license.

### RNA-seq.

RNA was harvested from mock-infected and high- or low-MOI SARS-CoV-2-infected cultures (*n* = 6) using TRIzol with gentle pipetting to recover the cells. Total RNA was extracted using the Zymo Direct-zol DNA/RNA miniprep kit according to the manufacturer’s protocol. The quality of the isolated RNA was assessed with a Qubit 2.0 fluorometer (Thermo Fisher Scientific), and RNA integrity was checked using a TapeStation system (Agilent Technologies). mRNA sequencing libraries were prepared by Genewiz. The RNA sequencing library was prepared using the NEBNext Ultra II RNA library prep kit for Illumina according to the manufacturer’s instructions (New England BioLabs). Briefly, mRNAs were initially enriched with oligo(dT) beads. Enriched mRNAs were fragmented for 15 min at 94°C. First-strand and second-strand cDNAs were subsequently synthesized. cDNA fragments were end repaired and adenylated at the 3′ ends, and universal adapters were ligated to cDNA fragments, followed by index addition and library enrichment by PCR with limited cycles. The sequencing library was validated on the Agilent TapeStation system (Agilent Technologies) and quantified by using a Qubit 2.0 fluorometer (Thermo Fisher Scientific) as well as by quantitative PCR (Kapa Biosystems). The sequencing libraries were multiplexed and clustered onto a flow cell. After clustering, the flow cell was loaded onto the Illumina HiSeq instrument according to the manufacturer’s instructions. The samples were sequenced using a 2× 150-bp paired-end (PE) configuration. Image analysis and base calling were conducted by HiSeq Control software (HCS). Raw sequence data (.bcl files) generated from Illumina HiSeq were converted into fastq files and demultiplexed using Illumina bcl2fastq 2.17 software. One mismatch was allowed for index sequence identification.

### Genome-wide DNA methylation profiling analysis.

DNA was isolated from mock-infected and SARS-CoV-2-infected primary astrocyte cultures (*n* = 6) and brain organoids (*n* = 3). Total DNA was extracted using the Zymo Direct-zol DNA/RNA miniprep kit according to the manufacturer’s protocol. DNA methylation profiling of primary astrocyte cultures was performed by utilizing the EZ DNA methylation kit for bisulfite conversion and the Zymo-Seq Reduced representation bisulfite sequencing (RRBS) library preparation kit. The experimental protocol steps were as follows: (i) DNA digestion using the MspI restriction enzyme, which cuts DNA at its recognition site (C↓CGG), independent of the CpG methylation status; (ii) end repair and ligation of adapters for Illumina sequencing; (iii) bisulfite conversion using the lightning conversion reaction at 98°C for 8 min and 54°C for 1 h; (iv) index primer amplification by PCR for 16 cycles; and (v) sequencing on the NovaSeq platform. Adapters were trimmed with TrimGalore and aligned with bismark, yielding on average of 2.5 million CpGs per sample with 10× coverage. Differential methylation analysis was done using methylkit to perform pairwise comparisons of mock versus an MOI of 1, mock versus an MOI of 5, and an MOI of 1 versus an MOI of 5. Sites with an FDR of <0.05 and a methylation difference of at least 10% were considered differentially methylated. DNA methylation profiling of brain organoids was performed by utilizing the EZ DNA methylation kit for bisulfite conversion, and bisulfite-converted DNA samples were randomly assigned to a chip well on the Infinium human methylation Infinium MethylationEPIC BeadChip (EPIC) bead chip, amplified, hybridized onto the array, stained, washed, and imaged with the Illumina iSCAN SQ instrument to obtain raw image intensities and IDAT files. EPIC array data were loaded, preprocessed, and analyzed in the R statistical programming language. Methylation β-values ranging from 0 to 1 (corresponding to the ratio of the unmethylated to the methylated signal intensity) for each sample were normalized using the BMIQ function implemented in the ChAMP pipeline. Differential methylation analysis was conducted on the site level using linear models employed in the limma R package. Sites were identified as significant (*P* < 0.05) and filtered for sites with absolute methylation differences of >5% (Δβ-value) between groups. Differentially methylated probes were annotated using the EPIC array R package annotation IlluminaHumanMethylationEPICanno.ilm10b4.hg19.

### NanoString GeoMx DSP RNA assays.

Slides were prepared according to the manual RNA slide preparation protocol in the GeoMx DSP slide preparation user’s manual (MAN-10115-03 for software v2.1; NanoString). Briefly, slides were baked at 60°C for 45 min. Deparaffinization and rehydration were performed in xylene 3 times for 5 min each, 100% ethanol twice for 5 min each, 95% ethanol once for 5 min, and 1× PBS once for 5 min. Antigen retrieval was performed in 1× Tris-EDTA (pH 9.0) (catalog number SRE0063; Sigma-Aldrich) in a Tinto Retriever pressure cooker for 15 min at 100°C. Thereafter, the slides were washed with 1× PBS for 5 min. Tissue RNA targets were exposed by incubating the slides with 1 mg/mL proteinase K (catalog number 2546; Ambion) in PBS for 15 min at 37°C. Slides were washed in 1× PBS for 5 min and then immediately placed into 10% neutral buffered formalin (NBF) for 5 min, followed by incubation in NBF stop buffer (1.48 M Tris base, 563 mM glycine) twice for 5 min each and in PBS once for 5 min. *In situ* hybridizations were performed with the GeoMx whole-transcriptome atlas (WTA) panel (18,335 targets). One at a time, the slides were dried of excess 1× PBS, set in a hybridization chamber lined with Kimwipes, wetted with 2× SSC (1× SSC is 0.15 M NaCl plus 0.015 M sodium citrate), and covered with 200 μL of the prepared probe hybridization solution. HybriSlips (catalog number 714022; Grace Biolabs) were gently applied to each slide, and each slide was incubated at 37°C overnight. The HybriSlips were removed by dipping the slides in 2× SSC (catalog number S6639; Sigma-Aldrich)–0.1% Tween 20. To remove unbound probes, the slides were washed twice in 50% formamide (catalog number AM9342; Thermo Fisher)–2× SSC at 37°C for 25 min, followed by two washes in 2× SSC for 2 min. Slides were blocked in 200 μL buffer W (NanoString), placed in a humidity chamber, and incubated at room temperature for 30 min. A morphology marker solution was prepared, containing SARS-CoV-2 spike (NanoString) and 187 μL buffer W, for a total volume of 220 μL/slide. One at a time, the slides were dried of excess buffer W, set in a humidity chamber, covered with 200 μL of the morphology marker solution, and left to incubate at room temperature for 2 h. The slides were washed twice with 2× SSC for 5 min and immediately loaded onto the NanoString DSP instrument.

### Statistical analyses.

All data are shown as means ± standard errors of the means (SEM). Statistical analyses for the qPCR experiments were performed using GraphPad Prism 9 software and included one-way analysis of variance (ANOVA). Statistical analysis for the immunofluorescence cell counts was performed using Student’s *t* test.

### Resource availability.

Further information and requests for resources and reagents should be directed to and will be fulfilled by the corresponding author.

### Material availability.

This study did not generate new unique reagents. The Table S3 contains a list of all materials used for this study.

### Data availability.

Raw RNA-seq data are available at the Gene Expression Omnibus (GEO) under accession number GSE198722. The data that support the findings of this study are available from the corresponding author upon request.

10.1128/mbio.02308-22.9 TABLE S3 Materials used in this study. Download Table S3, DOCX file, 0.02 MB. Copyright © 2022 Kong et al. 2022 Kong et al. https://creativecommons.org/licenses/by/4.0/ This content is distributed under the terms of the Creative Commons Attribution 4.0 International license.
