# Supplementary figures and images for: Neuropilin-1 Mediates SARS-CoV-2 Infection of Astrocytes in Brain Organoids, Inducing Inflammation Leading to Dysfunction and Death of Neurons
Source: mBio. 2022 Oct 31;13(6):e02308-22. doi: 10.1128/mbio.02308-22 (PMC9765283; doi:10.1128/mbio.02308-22)

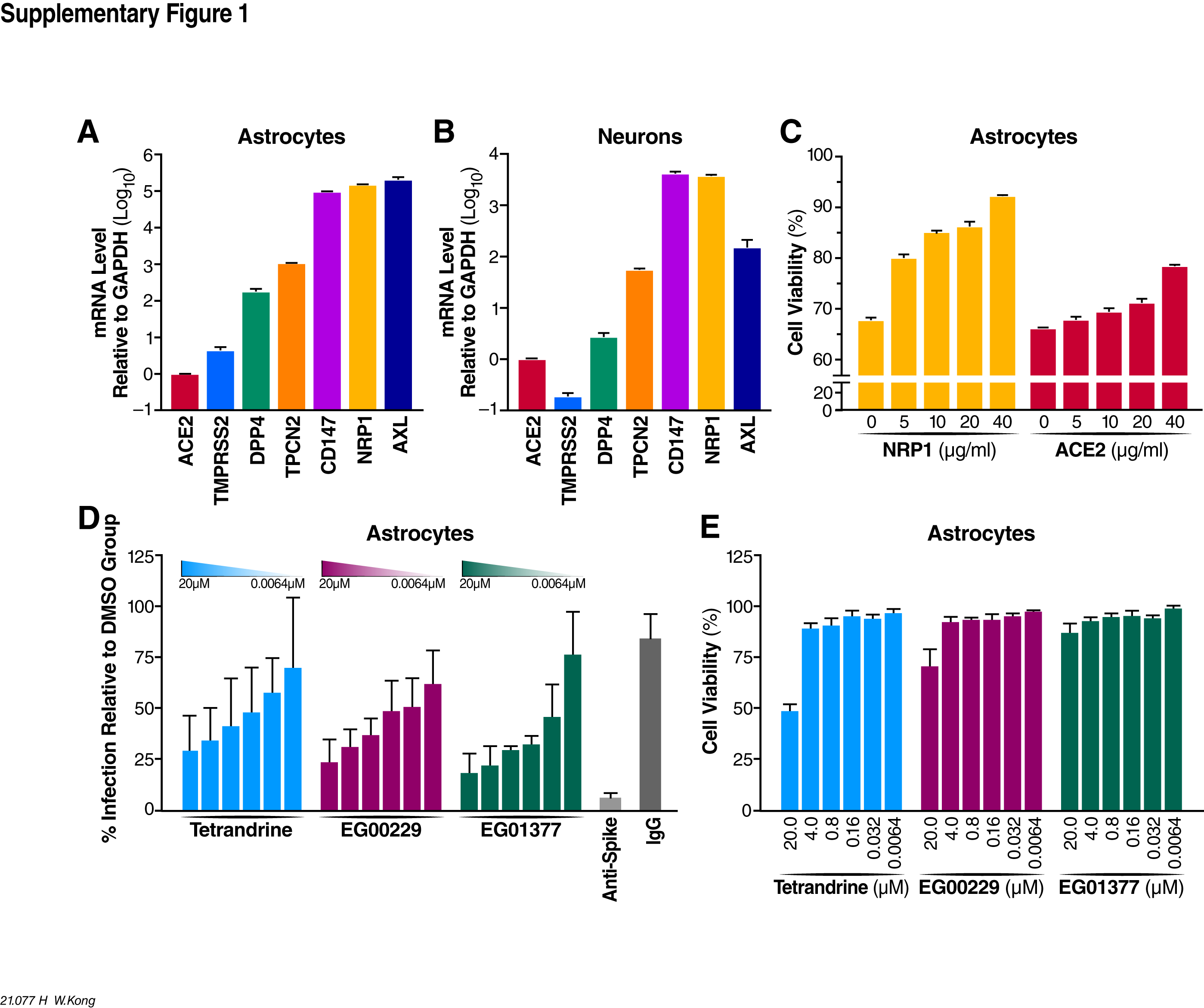

Supplement: FIG S1 [file mbio.02308-22-s0001.tif]

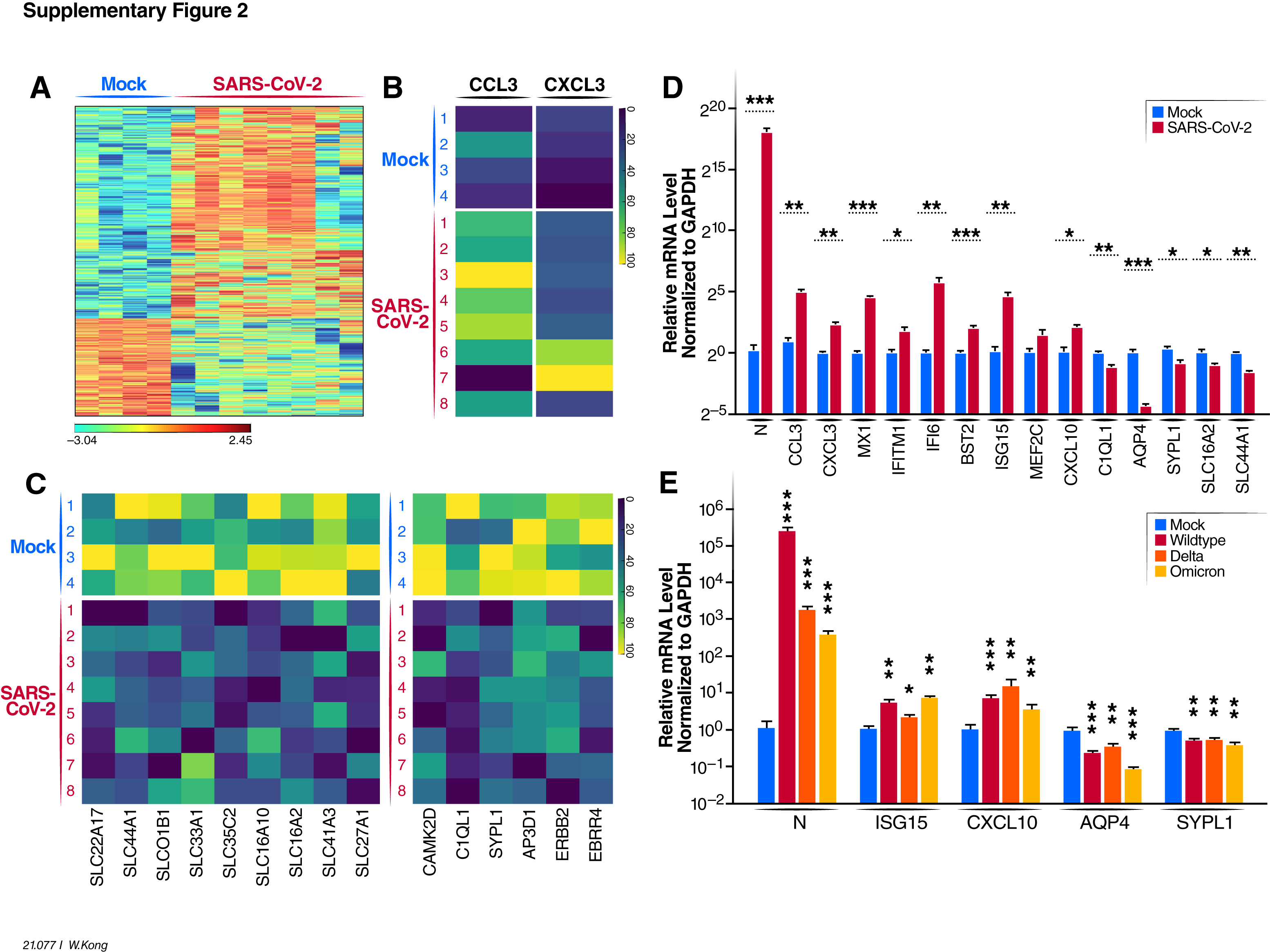

Supplement: FIG S2 [file mbio.02308-22-s0002.tif]

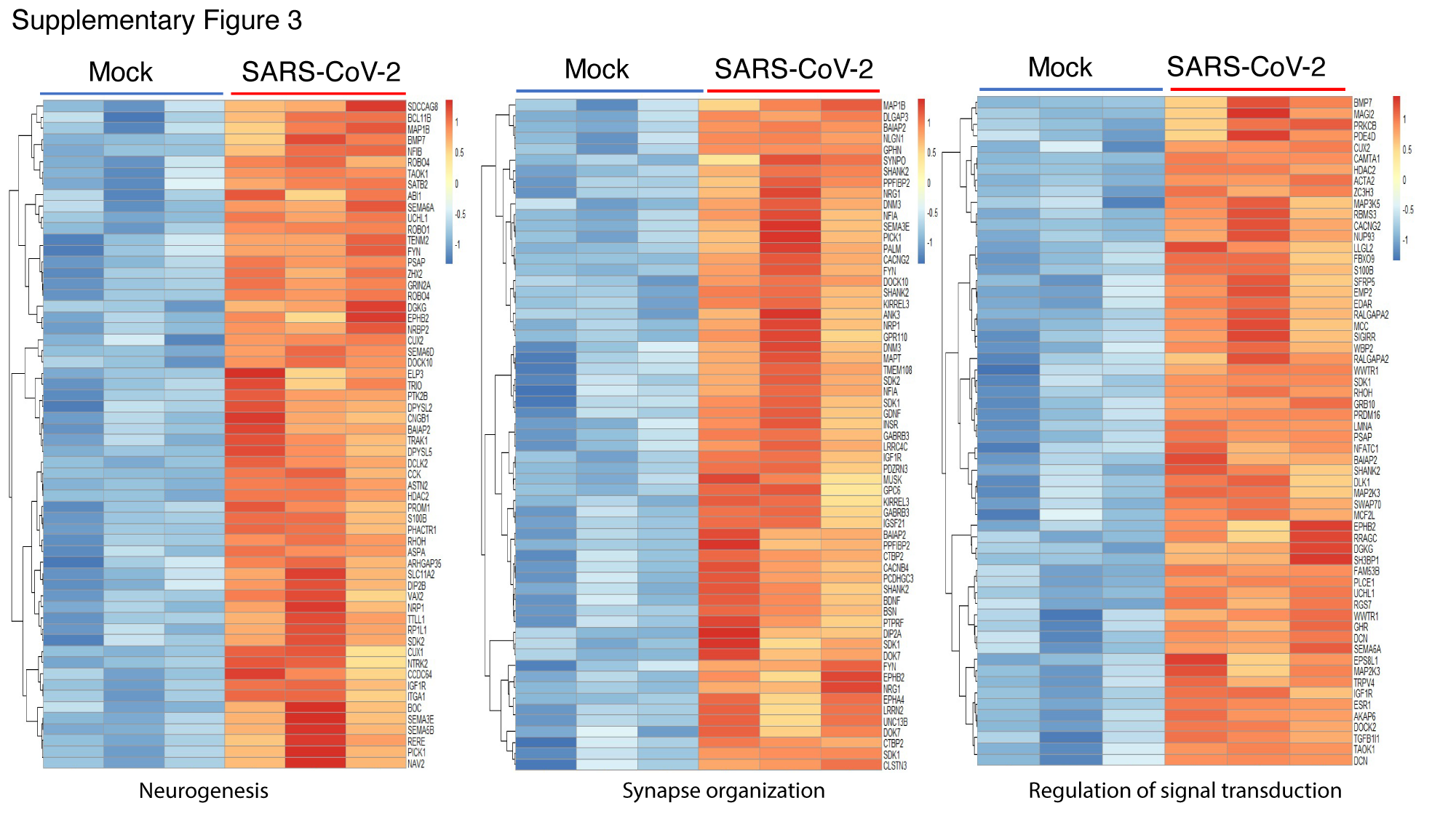

Supplement: FIG S3 [file mbio.02308-22-s0003.tif]

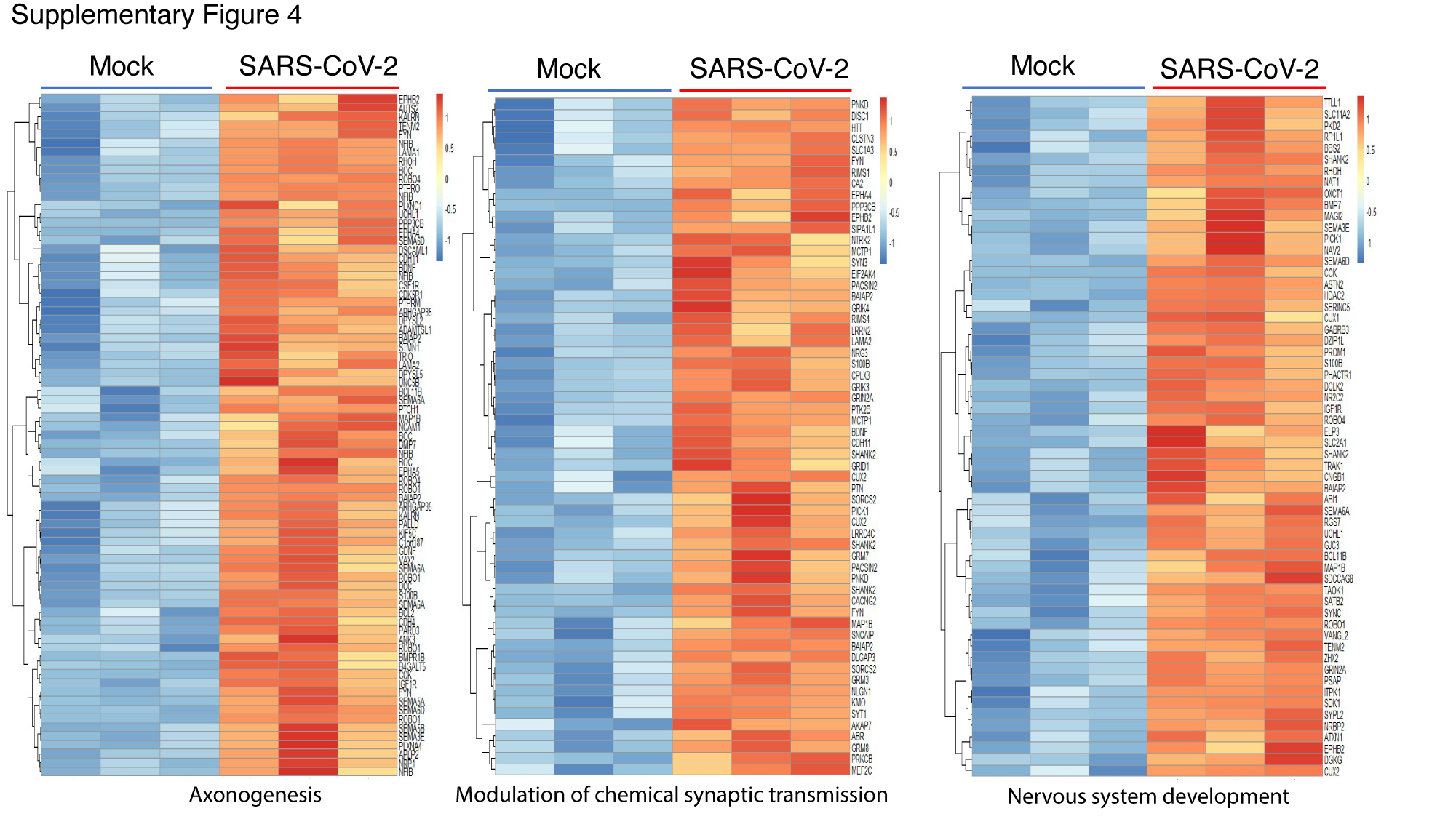

Supplement: FIG S4 [file mbio.02308-22-s0004.tif]

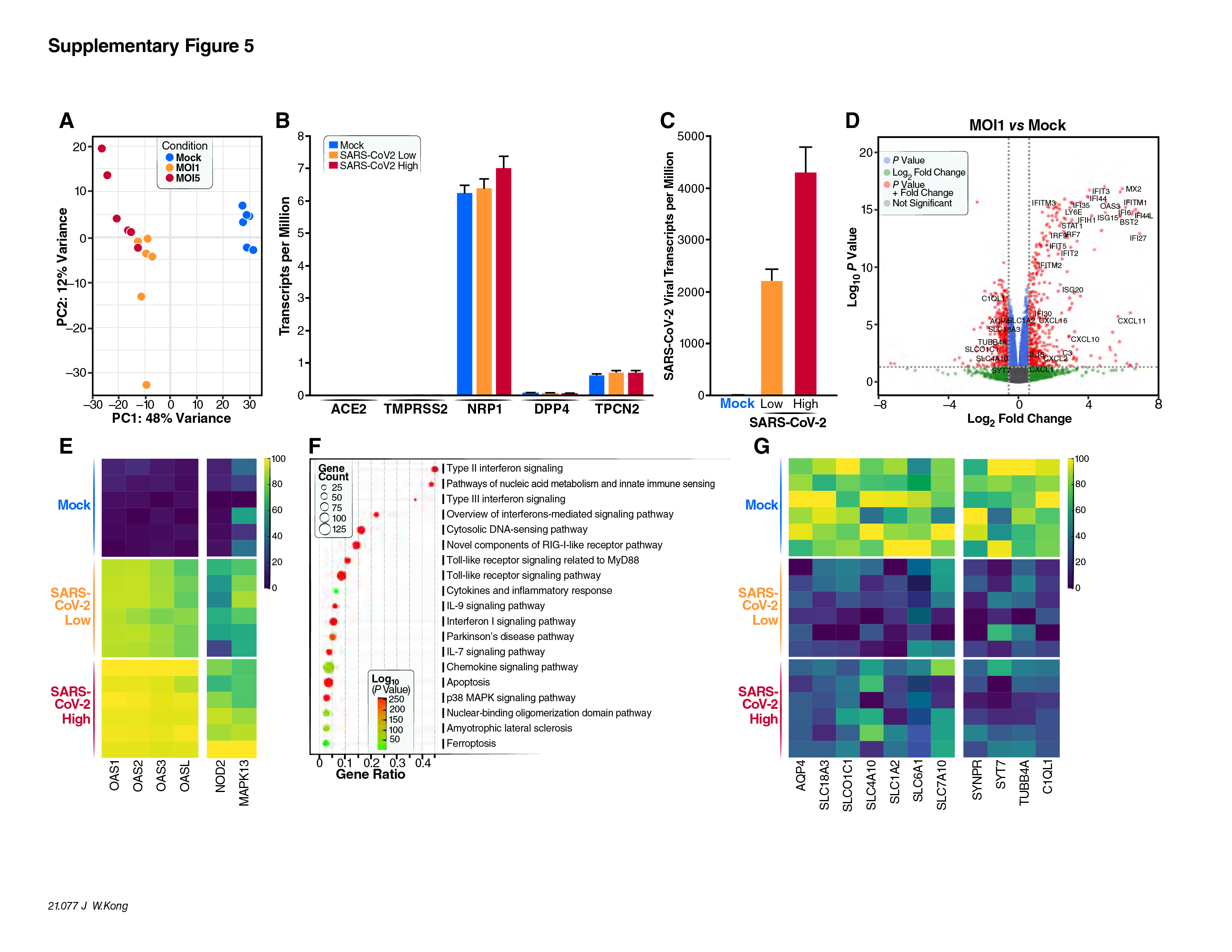

Supplement: FIG S5 [file mbio.02308-22-s0005.tif]

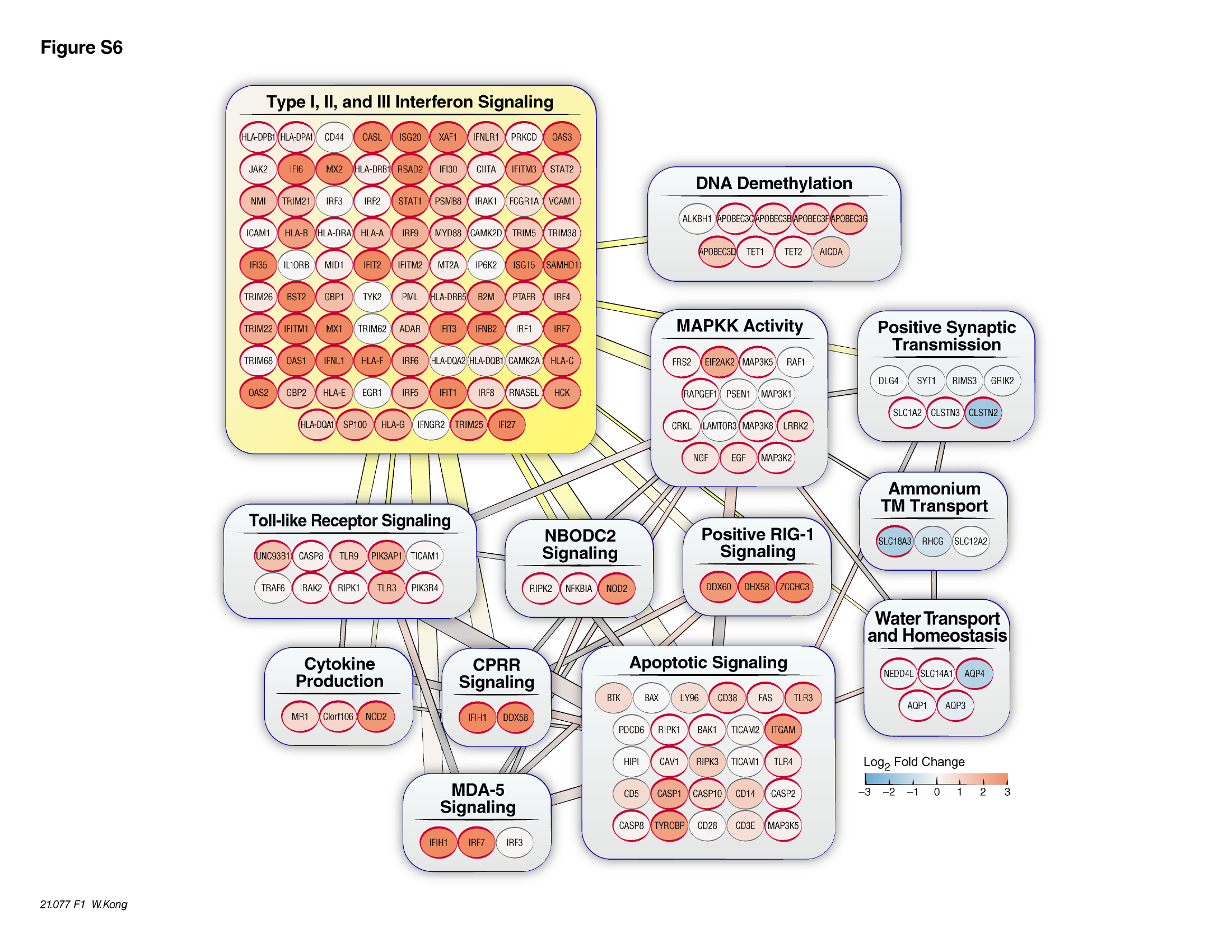

Supplement: FIG S6 [file mbio.02308-22-s0006.tif]
