# Supplementary material for: Neuropilin-1 Mediates SARS-CoV-2 Infection of Astrocytes in Brain Organoids, Inducing Inflammation Leading to Dysfunction and Death of Neurons
Source: mBio. 2022 Oct 31;13(6):e02308-22. doi: 10.1128/mbio.02308-22 (PMC9765283; doi:10.1128/mbio.02308-22)
Supplement: TABLE S1 [file mbio.02308-22-s0007.docx]

| Genes | Natural function | Role in SARS-CoV-2 infection |
| --- | --- | --- |
| ACE2 | Cell surface receptor and peptidase that cleaves angiotensin II and other peptide hormones. | Binding with spike protein, mediating cell entry ^1-3^. |
| TMPRSS2 | Androgen-responsive serine protease. | Primes spike for entry, facilitating viral entry and activation ^3^. |
| NRP1 | Cell surface receptor is involved in the development of the cardiovascular system, in angiogenesis, in the formation of certain neuronal circuits and in organogenesis outside the nervous system. | NRP1 binds to furin-cleaved S1 fragment of the spike protein and facilitates SARS-CoV-2 cell entry and infectivity ^4,5^. |
| AXL | It plays a role in various processes such as endothelial cell survival during acidification by preventing apoptosis, optimal cytokine signaling during human natural killer cell development. | AXL interacts with spike and promotes viral entry ^6^.  AXL does not bind to spike, mediates enhancement of SARS-CoV-2 infection through interactions with virion-associated phosphatidylserine in an ACE2-dependent manner ^7^. |
| DPP4 | Widely expressed enzyme transducing actions through an anchored transmembrane molecule and a soluble circulating protein. | DPP4 interacts with spike and promotes viral entry ^8^.  DPP4 is not a receptor and does not promote viral entry ^1,9^. |
| CD147 | The receptor for cyclophilins, S100A9, platelet glycoprotein VI, the rod-derived cone viability factor. | CD147 interacts with spike and promotes viral entry^10^.  CD147 does not bind with spike ^11,12^. |
| TPCN2 | A lysosomal non-selective Na^+^/Ca^2+^ channel. | Essential in the endocytosis of SARS-CoV-2 and Ebola virus ^13,14^ . |
| CTSL | A lysosomal cysteine protease enzyme with endopeptidase activity | CTSL functionally cleaved the SARS-CoV-2 spike protein and enhanced virus entry^15^. |
| Furin | A protease known for cleaving inactive precursor proteins into their biologically active product. | Cleaves spike protein, facilitating viral entry ^16,17^. |

Table S1: The role of host factors in SARS-CoV-2 infection.

1. Zhou, P., Yang, X.L., Wang, X.G., Hu, B., Zhang, L., Zhang, W., Si, H.R., Zhu, Y., Li, B., Huang, C.L., et al. (2020). A pneumonia outbreak associated with a new coronavirus of probable bat origin. Nature *579*, 270-273. 10.1038/s41586-020-2012-7.

2. Lan, J., Ge, J., Yu, J., Shan, S., Zhou, H., Fan, S., Zhang, Q., Shi, X., Wang, Q., Zhang, L., and Wang, X. (2020). Structure of the SARS-CoV-2 spike receptor-binding domain bound to the ACE2 receptor. Nature *581*, 215-220. 10.1038/s41586-020-2180-5.

3. Hoffmann, M., Kleine-Weber, H., Schroeder, S., Kruger, N., Herrler, T., Erichsen, S., Schiergens, T.S., Herrler, G., Wu, N.H., Nitsche, A., et al. (2020). SARS-CoV-2 Cell Entry Depends on ACE2 and TMPRSS2 and Is Blocked by a Clinically Proven Protease Inhibitor. Cell *181*, 271-280 e278. 10.1016/j.cell.2020.02.052.

4. Daly, J.L., Simonetti, B., Klein, K., Chen, K.E., Williamson, M.K., Anton-Plagaro, C., Shoemark, D.K., Simon-Gracia, L., Bauer, M., Hollandi, R., et al. (2020). Neuropilin-1 is a host factor for SARS-CoV-2 infection. Science *370*, 861-865. 10.1126/science.abd3072.

5. Cantuti-Castelvetri, L., Ojha, R., Pedro, L.D., Djannatian, M., Franz, J., Kuivanen, S., van der Meer, F., Kallio, K., Kaya, T., Anastasina, M., et al. (2020). Neuropilin-1 facilitates SARS-CoV-2 cell entry and infectivity. Science *370*, 856-860. 10.1126/science.abd2985.

6. Wang, S., Qiu, Z., Hou, Y., Deng, X., Xu, W., Zheng, T., Wu, P., Xie, S., Bian, W., Zhang, C., et al. (2021). AXL is a candidate receptor for SARS-CoV-2 that promotes infection of pulmonary and bronchial epithelial cells. Cell Res *31*, 126-140. 10.1038/s41422-020-00460-y.

7. Bohan, D., Ert, H.V., Ruggio, N., Rogers, K.J., Badreddine, M., Aguilar Briseno, J.A., Rojas Chavez, R.A., Gao, B., Stokowy, T., Christakou, E., et al. (2021). Phosphatidylserine Receptors Enhance SARS-CoV-2 Infection: AXL as a Therapeutic Target for COVID-19. bioRxiv. 10.1101/2021.06.15.448419.

8. Li, Y., Zhang, Z., Yang, L., Lian, X., Xie, Y., Li, S., Xin, S., Cao, P., and Lu, J. (2020). The MERS-CoV Receptor DPP4 as a Candidate Binding Target of the SARS-CoV-2 Spike. iScience *23*, 101400. 10.1016/j.isci.2020.101400.

9. Xie, X., Muruato, A.E., Zhang, X., Lokugamage, K.G., Fontes-Garfias, C.R., Zou, J., Liu, J., Ren, P., Balakrishnan, M., Cihlar, T., et al. (2020). A nanoluciferase SARS-CoV-2 for rapid neutralization testing and screening of anti-infective drugs for COVID-19. Nat Commun *11*, 5214. 10.1038/s41467-020-19055-7.

10. Wang, K., Chen, W., Zhang, Z., Deng, Y., Lian, J.Q., Du, P., Wei, D., Zhang, Y., Sun, X.X., Gong, L., et al. (2020). CD147-spike protein is a novel route for SARS-CoV-2 infection to host cells. Signal Transduct Target Ther *5*, 283. 10.1038/s41392-020-00426-x.

11. Shilts, J., Crozier, T.W.M., Greenwood, E.J.D., Lehner, P.J., and Wright, G.J. (2021). No evidence for basigin/CD147 as a direct SARS-CoV-2 spike binding receptor. Sci Rep *11*, 413. 10.1038/s41598-020-80464-1.

12. Ragotte, R.J., Pulido, D., Donnellan, F.R., Hill, M.L., Gorini, G., Davies, H., Brun, J., McHugh, K., King, L.D.W., Skinner, K., et al. (2021). Human Basigin (CD147) Does Not Directly Interact with SARS-CoV-2 Spike Glycoprotein. mSphere *6*, e0064721. 10.1128/mSphere.00647-21.

13. Ou, X., Liu, Y., Lei, X., Li, P., Mi, D., Ren, L., Guo, L., Guo, R., Chen, T., Hu, J., et al. (2020). Characterization of spike glycoprotein of SARS-CoV-2 on virus entry and its immune cross-reactivity with SARS-CoV. Nat Commun *11*, 1620. 10.1038/s41467-020-15562-9.

14. Sakurai, Y., Kolokoltsov, A.A., Chen, C.C., Tidwell, M.W., Bauta, W.E., Klugbauer, N., Grimm, C., Wahl-Schott, C., Biel, M., and Davey, R.A. (2015). Ebola virus. Two-pore channels control Ebola virus host cell entry and are drug targets for disease treatment. Science *347*, 995-998. 10.1126/science.1258758.

15. Zhao, M.M., Yang, W.L., Yang, F.Y., Zhang, L., Huang, W.J., Hou, W., Fan, C.F., Jin, R.H., Feng, Y.M., Wang, Y.C., and Yang, J.K. (2021). Cathepsin L plays a key role in SARS-CoV-2 infection in humans and humanized mice and is a promising target for new drug development. Signal Transduct Target Ther *6*, 134. 10.1038/s41392-021-00558-8.

16. Papa, G., Mallery, D.L., Albecka, A., Welch, L.G., Cattin-Ortola, J., Luptak, J., Paul, D., McMahon, H.T., Goodfellow, I.G., Carter, A., et al. (2021). Furin cleavage of SARS-CoV-2 Spike promotes but is not essential for infection and cell-cell fusion. PLoS Pathog *17*, e1009246. 10.1371/journal.ppat.1009246.

17. Xia, S., Lan, Q., Su, S., Wang, X., Xu, W., Liu, Z., Zhu, Y., Wang, Q., Lu, L., and Jiang, S. (2020). The role of furin cleavage site in SARS-CoV-2 spike protein-mediated membrane fusion in the presence or absence of trypsin. Signal Transduct Target Ther *5*, 92. 10.1038/s41392-020-0184-0.
