# Supplementary material for: Neuropilin-1 Mediates SARS-CoV-2 Infection of Astrocytes in Brain Organoids, Inducing Inflammation Leading to Dysfunction and Death of Neurons
Source: mBio. 2022 Oct 31;13(6):e02308-22. doi: 10.1128/mbio.02308-22 (PMC9765283; doi:10.1128/mbio.02308-22)
Supplement: TABLE S2 [file mbio.02308-22-s0008.docx]

| Gene | Primer sequence (5’-3’) Forward | Primer sequence (5’-3’) Reverse |
| --- | --- | --- |
| NRP1 | GCGGCTCACAAAGAATAAGC | ATCCACCAAAACCAACCAAA |
| TPCN2 | AAGGTGCGTTCCTATGGCAGTG | GCAGAAACGGAGACTGGTACTC |
| ACE2 | GGGATCAGAGATCGGAAGAAGAAA | AGGAGGTCTGAACATCATCAGTG |
| TMPRSS2 | ACTCTGGAAGTTCATGGGCAG | TGAAGTTTGGTCCGTAGAGGC |
| CTSL | GAAAGGCTACGTGACTCCTGTG | CCAGATTCTGCTCACTCAGTGAG |
| AXL | GTTTGGAGCTGTGATGGAAGGC | CGCTTCACTCAGGAAATCCTCC |
| FURIN | GCCACATGACTACTCCGCAGAT | TACGAGGGTGAACTTGGTCAGC |
| GAPDH | GCCTCTTGTCTCTTAGATTTGGTC | TAGCACTCACCATGTAGTTGAGGT |
| DPP4 | CAAAAACACAGCAAGGGTGA | TAACAGGGCAAGCTGATGTG |
| Spike(S) | CCTACTAAATTAAATGATCTCTGCTTTACT | CAAGCTATAACGCAGCCTGTA |
| [Nucleocapsid](https://www.rndsystems.com/products/recombinant-hcov-oc43-nucleocapsid-his-tag-protein-cf_10709-cv)(N) | GGGGAACTTCTCCTGCTAGAAT | CAGACATTTTGCTCTCAAGCTG |
| RdRp | CAAGTGGGGTAAGGCTAGACTTT | ACTTAGGATAATCCCAACCCAT |
| CD147 | GGCTGTGAAGTCGTCAGAACAC | ACCTGCTCTCGGAGCCGTTCA |
| ISG15 | CTCTGAGCATCCTGGTGAGGAA | AAGGTCAGCCAGAACAGGTCGT |
| MX1 | GGCTGTTTACCAGACTCCGACA | CACAAAGCCTGGCAGCTCTCTA |
| IFITM1 | GGCTTCATAGCATTCGCCTACTC | AGATGTTCAGGCACTTGGCGGT |
| IFI6 | TGATGAGCTGGTCTGCGATCCT | GTAGCCCATCAGGGCACCAATA |
| BST2 | TCTCCTGCAACAAGAGCTGACC | TCTCTGCATCCAGGGAAGCCAT |
| MEF2C | TCCACCAGGCAGCAAGAATACG | GGAGTTGCTACGGAAACCACTG |
| AQP4 | GCCATCATT GGAGCAGGAATCC | ACTCAACCAGGAGACCATGACC |
| SLC44A1 | CCTTGTTCCACGTAGCTGGCAA | GCCTTGCTCATTCTGAACAGGAC |
| C1QL | AGTATGTGGGCAGACCTCTGCA | AGTATGTGGGCAGACCTCTGCA |
| SLC16A2 | CAACATGCGAGTGTTCCGCCAA | AAGAGCACCCAGGTCTCCTTGA |
| CCL3 | ACTTTGAGACGAGCAGCCAGTG | TTTCTGGACCCACTCCTCACTG |
| CXCL3 | TTCACCTCAAGAACATCCAAAGTG | TTCTTCCCATTCTTGAGTGTGGC |
| SYPL1 | CTTTGGCTCTGTGACCAGTATGG | GATGGACTGTGTAGGCTGGTCT |

Table S2: Primers are used in RT-qPCR.
