# Supplementary material for: Neuropilin-1 Mediates SARS-CoV-2 Infection of Astrocytes in Brain Organoids, Inducing Inflammation Leading to Dysfunction and Death of Neurons
Source: mBio. 2022 Oct 31;13(6):e02308-22. doi: 10.1128/mbio.02308-22 (PMC9765283; doi:10.1128/mbio.02308-22)
Supplement: TABLE S3 [file mbio.02308-22-s0009.docx]

Table S3: List of materials used in this study.

| **REAGENT or RESOURCE** | | **SOURCE** | | **IDENTIFIER** |
| --- | --- | --- | --- | --- |
| Antibodies | | | | |
| Rabbit monoclonal anti-NRP1 | | Thermo Fisher Scientific | | Cat#MA5-32179 |
| Rabbit monoclonal anti-PAX6 | | Thermo Fisher Scientific | | Cat#42-6660 |
| Mouse monoclonal anti-NRP1 | | Protein Tech | | Cat#60067-1-Ig |
| Mouse monoclonal anti-Spike | | GeneTex | | Cat#GTX632604 |
| Rabbit monoclonal anti- Nucleocapsid | | GeneTex | | Cat#GTX635689 |
| Mouse monoclonal anti-MAP2 | | Millipore Sigma | | Cat#MAB3418 |
| Rabbit polyclonal anti-NESTIN | | Millipore Sigma | | Cat#ABD69 |
| Mouse monoclonal anti-β-Actin | | Millipore Sigma | | Cat#A5316 |
| Mouse monoclonal anti-TUJ1 | | BioLegend | | Cat#801201 |
| Chicken polyclonal anti- MAP2 | | EnCor | | Cat#CPCA-MAP2 |
| Chicken polyclonal anti- GFAP | | EnCor | | Cat#CPCA-GFAP |
| Rabbit polyclonal anti-GFAP | | Novus Biologicals | | Cat#NB300-141 |
| Goat polyclonal anti-ACE2 | | R & D | | Cat#AF933 |
| Goat polyclonal anti-AXL | | R & D | | Cat#AF154 |
| AlexaFluor 488, 647 goat anti-rabbit IgG (H+L) | | Life Technologies | | Cat#A11008, A2134003 |
| AlexaFluor 488, 647 goat anti-chicken IgG (H+L) | | Life Technologies | | Cat#A32931, A32933 |
| AlexaFluor 488, 568, goat anti-mouse IgG (H+L) | | Life Technologies | | Cat#A11001, A11031 |
| Vector® TrueVIEW® Autofluorescence Quenching Kit with DAPI | | VECTOR laboratories | | SP-8500-15 |
| Bacterial and virus strains | |  | |  |
| SARS-CoV-2/human/USA/USA-WA1/2020 (WA1) (BEI NR-52281), Delta B.1.617.2 (BEI NR-55611) and Omicron B.1.1.529. | | Gladstone Institutes | |  |
| SARS-CoV-2-Nluc | Xie et al.,2020 | | https://www.nature.com/articles/s41467-020-19055-7 | |
| Chemicals, peptides, and recombinant proteins | | | | |
| Matrigel | | Corning | | Cat#354234 |
| Lipofectamine™ RNAiMAX Transfection Reagent | | Life Technologies | | Cat#13778030 |
| Rock Inhibitor Y27632 | | Selleckchem | | Cat#S1049 |
| Gentle Cell Dissociation Reagent | | Stem Cell Technologies | | Cat#100-0485 |
| EG00229 | | MedChemExpress | | Cat#HY-10799 |
| EG01377 | | MedChemExpress | | Cat#HY-112151 |
| Tetrandrine | | Selleckchem | | Cat#S2403 |
| Doxycycline | | Selleckchem | | Cat# S5159 |
| BDNF | | Peprotech | | Cat# 450-02 |
| NT3 | | Peprotech | | Cat# 450-03 |
| Laminin | | Life Technologies | | Cat# 23017-015 |
| Critical commercial assays | | | | |
| Cerebral Organoid kit | | Stem Cell Technologies | | Cat#08570, 08571 |
| Quick-RNA Miniprep | | ZYMO RESEARCH | | Cat#R1054 |
| Direct-zol DNA/RNA Miniprep | | ZYMO RESEARCH | | Cat#R2080 |
| TRI Reagent | | ZYMO RESEARCH | | Cat#R2050-1-50 |
| QIAshredder | | QIAGEN | | Cat#79654 |
| AllPrep DNA/RNA Micro Kit | | QIAGEN | | Cat#80284 |
| iTaq Universal SYBR Green Supermix | | Bio-Rad | | Cat#1725121 |
| RNeasy Plus Micro Kit | QIAGEN | | Cat#74034 | |
| TUNEL Assay kit | | Life Technologies | | Cat# C10617 |
| RNeasy Mini Kit | | QIAGEN | | Cat#74104 |
| iScript™ cDNA Synthesis Kit | | Bio-Rad | | Cat#1708891 |
| EZ DNA Methylation Kit | | ZYMO RESEARCH | | Cat#D5001 |
| Zymo-Seq RRBS Library Kit | | ZYMO RESEARCH | | Cat#D5460 |
| Infinium Methylation EPIC BeadChip Kit | | Illumina | | Cat#WG-317-1001 |
| Experimental models: Cell lines | | | | |
| Human: WTC11 iPSC | | Gladstone institutes | | N/A |
| Human: HEK293T | | ATCC | | CRL-3216 |
| Human: Astrocytes | | ScienCell Research Laboratories | | Cat#1800 |
| Monkey: Vero-E6 | | ATCC | | CRL-1586 |
| Oligonucleotides | |  | |  |
| siRNA-ACE2 | | ThermoFisher Scientific | | Cat#4427037- s33964, 4427037- s33965 |
| siRNA-TMPRSS2 | | ThermoFisher Scientific | | Cat#4427037- s14236, 4427037- s14237 |
| siRNA-TPCN2 | | ThermoFisher Scientific | | Cat#AM51331- 37190, AM51331-104850 |
| siRNA-NRP1 | | ThermoFisher Scientific | | Cat#AM51331- 114375, 4390824- s16843 |
| siRNA-DPP4 | | ThermoFisher Scientific | | Cat#4427037- s4254, 4427037- s4255 |
| siRNA-CTSL | | ThermoFisher Scientific | | Cat#4427038-s3753,  4427038-s223364 |
| siRNA-CD147 | | ThermoFisher Scientific | | Cat#4427037-S2098, 4427037-S2099 |
| siRNA-AXL | | ThermoFisher Scientific | | Cat# 4427038-S1846,  4427038-S1847 |
| siRNA-negative control | | ThermoFisher Scientific | | Cat#AM4611, 4390843 |
| Software and algorithms | | | | |
| ImageJ | | Schneider et al., 2012 | | https://imagej.nih.gov/ij/ |
| GeoMx DSP | | NanoString | | https://www.nanostring.com/ |
| Graphpad Prism 9.0 | | Graphpad | | https://www.graphpad.com/ |
| CellProfiler | |  | | https://cellprofiler.org/ |
| Adobe Illustrator | | Adobe | | <https://www.adobe.com/products/illustrator.html> |
| Other | | | | |
|  | |  | |  |
|  | |  | |  |
|  | |  | |  |
|  | |  | |  |
